# Conservation of *k*-mer Composition and Correlation Contribution between Introns and Intergenic Regions of Animalia Genomes

**DOI:** 10.3390/genes9100482

**Published:** 2018-10-04

**Authors:** Aaron Sievers, Frederik Wenz, Michael Hausmann, Georg Hildenbrand

**Affiliations:** 1 Kirchhoff-Institute for Physics, Heidelberg University, INF 227, 69117 Heidelberg, Germany; Aaron.Sievers@stud.uni-heidelberg.de (A.S.); hausmann@kip.uni-heidelberg.de (M.H.); 2 Department of Radiation Oncology, Medical Faculty Mannheim, Universitätsmedizin Mannheim, Heidelberg University, Theodor-Kutzer-Ufer 1-3, 68167 Mannheim, Germany; Frederik.Wenz@medma.uni-heidelberg.de

**Keywords:** *k*-mer, sequence analysis, alignment-free, sequence patterns, Animalia, intron, intergenic region, tandem repeats, 3D conformation of DNA

## Abstract

In this study, we pairwise-compared multiple genome regions, including genes, exons, coding DNA sequences (CDS), introns, and intergenic regions of 39 Animalia genomes, including Deuterostomia (27 species) and Protostomia (12 species), by applying established *k*-mer-based (alignment-free) comparison methods. We found strong correlations between the sequence structure of introns and intergenic regions, individual organisms, and within wider phylogenetical ranges, indicating the conservation of certain structures over the full range of analyzed organisms. We analyzed these sequence structures by quantifying the contribution of different sets of DNA words to the average correlation value by decomposing the correlation coefficients with respect to these word sets. We found that the conserved structures within introns, intergenic regions, and between the two were mainly a result of conserved tandem repeats with repeat units ≤ 2 bp (e.g., (AT)_n_), while other conserved sequence structures, such as those found between exons and CDS, were dominated by tandem repeats with repeat unit sizes of 3 bp in length and more complex DNA word patterns. We conclude that the conservation between intron and intergenic regions indicates a shared function of these sequence structures. Also, the similar differences in conserved structures with known origin, especially to the conservation between exons and CDS resulting from DNA codons, indicate that *k*-mer composition-based functional properties of introns and intergenic regions may differ from those of exons and CDS.

## 1. Introduction

The conservation (within a wide phylogenetic range) of sequence structures within certain genome regions, like genes, exons, and especially coding DNA sequences (CDS) is out of the question. For DNA structures conserved between all known lifeforms (including viruses) within these regions, its information-storing capacity of the chemical structure of proteins, known as amino acid codons, has been known for many years [1]. At first declared as useless junk DNA [2,3], the existence of conserved structures within *non-coding regions* (defined as regions not coding for proteins, e.g., introns and intergenic regions) is also not a very new discovery [4,5]. Conserved structures found within introns can range from the sequences of individual introns of related genes [4] to global intron sequence structures, found using powerful, alignment-free methods [5,6,7]. Likewise, intergenic regions (defined as the regions between genes, excluding introns, 5′ untranslated regions (UTR), 3′UTR, and known structural elements like centromeres), have, in recent years, been found to harbor interesting and conserved sequence structures. The first hints at a functional connection between introns and intergenic regions in Animalia, namely a correlation between the size of the two regions, was found in [8]. Unlike the analysis and results shown in this article, the focus in that study was not on finding conserved sequence structures within one genome region, but instead, whether conserved patterns between (two or more) such regions (e.g., between exons and introns) could be found.

When trying to search for conserved sequence structures between complete genome sequences or regions of a comparable order of magnitude in size, such as regions consisting of all genes, introns, or intergenic regions with a large number of organisms, problems to face are those of large datasets and limited computational power. Additionally, between two such regions, and especially between non-coding regions, one cannot expect to find long, linearly conserved DNA sequences, as known to be present within exons and CDS, which can be easily pairwise aligned, using established alignment algorithms. Accordingly, standard tools like the NCBI Basic Local Alignment Search Tool (BLAST) [9], which deliver reliable results for the comparison of genes at the cost of relatively high consumption of computational resources, cannot be effectively used to face this task [10]. Fortunately, more recently developed alignment-free, computational, and much less complex algorithms were developed and proven to produce just as reliable results if used patiently [5,11,12]. We decided to use a quite simplistic but powerful method called *k*-mer-analysis [12] to pairwise-compare genome regions of a wide phylogenetic range for 39 Animalia organisms, with completely sequenced and assembled genome sequences. The *k*-mer-analysis delivers a quantification of correlation (using Pearson correlation coefficient [13]) between sequence structures found within pre-defined regions (e.g., introns, exons). If a significantly high correlation is found within a wide phylogenetic range of organisms, this can be interpreted as conservation of such structures within this phylogenetic range. While a standard *k*-mer-analysis is sufficient to discover conservation of sequence structures, it does not deliver information about the conserved sequence patterns responsible for this conservation; therefore, we went one step further and additionally analyzed the results by performing a decomposition of the correlation coefficient (see Section 2.2 for details) to quantify the contribution of different DNA words (e.g., X_n_, (XY)_n_ with X,Y ∈{A,C,G,T}). These lists of DNA words were then compared between the genome regions and between the organisms.

## 2. Materials and Methods

For our analysis, we used publicly available unmasked nucleotide sequences from the NCBI website, in FASTA and GenBank format (Table 1) [14]. Since using high-quality datasets of analyzed sequence regions is essential for our method to produce reliable results, and since we are also interested in intergenic regions, only organisms with completely sequenced and assembled chromosomes were used for the analysis shown in this article. Because Mammalia genomes would have been overrepresented without an additional limitation, we decided to limit their number to the 13 genomes listed in Table 1. This strict selection criterion limited the number of available genome sequences, but we concluded that 39 organisms are a sufficiently large set to produce reliable results. We used unmasked versions of the genomes to limit prior knowledge and prior computation of the sequences to a minimum. Low-complexity regions, especially short tandem repeats (STRs) and DNA satellites, could also potentially be conserved sequence patterns resulting in high or low correlation between different regions, as they were already known to show peculiar *k*-mer patterns within certain genome sequences [12].

### 2.1. *k*-mer-Analysis

In this study, *k*-mer analysis of a DNA sequence is defined as being the extraction and counting of every DNA word with length *k* (*k* bases along one strand), using a sliding window approach [15] to eliminate the influence of an otherwise arbitrary chosen starting point. Therefore, for every *k* we extracted one word for every position within the analyzed sequence. In this work, we have chosen word lengths of 1 ≤ *k* ≤ 11. We used a limit of *k* = 11 because computation times and used resources (e.g., used memory) increase with the word length *k,* and *k* = 11 seemed to be a reasonable limit to maintain acceptable computation times. Later on, we focused our analysis to *k* = 7 and *k* = 11 because earlier theoretical studies have shown that these values are high enough to extract a reasonable amount of information for sequences in the length regimes we are analyzing [16]. In addition, the results shown later in this article suggest that at least two values of *k* should be analyzed, what we used also as an upper limit in order to keep computational times low.

The result of such a *k*-mer analysis of a single sequence, which refers to the frequencies/content (normalized to 1) of each DNA word of length *k*, is called the associated *k*-mer spectrum. Such a *k*-mer spectrum can also be interpreted as a 4*^k^* dimensional vector, and as such, vector distances can be applied to compare two or more *k*-mer spectra in order to calculate a value which can be interpreted as a measurement of similarity between associated DNA sequences. For a better normalization, the Pearson correlation function [13] was used over the Euclidean distance. Using the Spearman correlation function [17] to prevent issues due to extreme values/content did not result in any significant advantages. Simulations based on randomly shuffled sequences (Markov models) have proven that for *k* = 7 and *k* = 10, values above 0.1 are very improbable results of randomness (see Appendix); thus, correlation values above 0.1 are considered significant.

To prevent confusion when DNA words of different lengths are discussed, we will always use a slash if two (or more) separated sequences are meant. For example, A/C means the two different lengths of the *k* = 1 words with one A and C. AC means the single *k* = 2 word, consisting of one A followed by one C. This method was formerly applied and described in [12]. Similar methods were developed and successfully used for many different tasks [5,7,11,15,18]. We have chosen this method over other alignment-free methods because it allows calculation of the contribution of arbitrary DNA word sets to correlation values by a correlation decomposition (see Section 2.3). A review of other *k*-mer-based methods can be found in [19].

### 2.2. *k*-mer Analysis on Genome Regions

We analyzed six different genome sequence regions (Table 2) by excluding/masking every part of the genome sequences (more precisely, every individual chromosome sequence) that was not part of that region before applying the *k*-mer analysis (see Section 2.1). This procedure could be interpreted as applying a componentwise sum of the *k*-mer-spectra vector representation (see Section 2.1 for explanation) over the parts of the sequence which are also a part of a respective region (e.g., over all genes for genes) weighted by their length to obtain a summary, or in some sense, an average *k*-mer-spectrum of all parts of the respective region (e.g., a summary *k*-mer spectrum representing all introns of an organism). The regions were defined based on the regions marked within the GenBank [14] files used.

To quantize the conservation within such a region, based on pairwise-calculated correlation values, we computed the mean correlation values by summing over the correlation values between all pairs of analyzed organisms. The error bars shown on corresponding figures are defined by the standard error of the mean (assuming normal distribution of the underlying values).

### 2.3. Correlation Decomposition

We used a method called correlation decomposition to decompose the Pearson correlation coefficient [13] into a sum, where each summand represents the contribution of a pre-defined set of DNA words to the correlation coefficient. We used this method over other approaches (e.g., the Euclidean distance between word counts) to produce values directly connected to the contribution of respective DNA words to the observed correlation values. The possibility to perform such decompositions was a result of the linearity of the Pearson correlation coefficient [13] with respect to individual vector dimensions associated with DNA words, and was thus another motivation for the use of this correlation function. The definition of Pearson Correlation [13] we are using within this article can be written as follows:

(1) $$\;r\left(x,y\right)=\;N\left(x,y\right)\sum_{i=1}^{n}\left(x_{i}-\bar{x}\right)\left(y_{i}-\bar{y}\right)\;$$

with *x* and *y* as a 4*^k^* dimensional vector representation of corresponding *k*-mer spectra (see Section 2.1), and accordingly, the number of components given by *n* = 4*^k^*. The normalization factor *N*(*x*,*y*) is defined as:

(2) $$\;N\left(x,y\right)={\left(\sqrt{\sum_{i=1}^{n}{\left(x_{i}-\bar{x}\right)}^{2}}\sqrt{\sum_{i=1}^{n}{\left(y_{i}-\bar{y}\right)}^{2}}\right)}^{-1}\;\;$$

The mean values $\bar{x}$ and $\bar{y}$ are both equal to 1/*n* = 4^−*k*^ since the components of *x* and *y* represent word contents which sum up to 1 ($\sum_{i=1}^{n}x_{i}=\;\sum_{i=1}^{n}y_{i}$ = 1) and the number of words is given by 4*^k^*. Thus, Equation (1) reduces to:

(3) $$\;r\left(x,y\right)=\;N\left(x,y\right)\sum_{i=1}^{4^{k}}\left(x_{i}-4^{-k}\right)\left(y_{i}-4^{-k}\right)\;$$

Omitting the normalization constant, which is independent of the index *i* and therefore only a constant factor (see Equation (1)), every index *i* of the sum in Equation (3) corresponds to the contribution of exactly one *k*-mer word to the correlation value *r*(*x*,*y*). Accordingly, one can decompose the sum in Equation (3) and thus the correlation value *r*(*x*,*y*) into the sum of different summands representing contributions of different sets of *k*-mer words (Equation (4)). In general, *S_j_* can be any set of *k*-mer words as long as all existing words of a given length are part of exactly one such set, *S_j_.* This means that the union of all sets yields to the full set of all *k*-mer words of a given length, and every word is only a member of one set, *S_j_* (e.g., *S*_1_ = {A, T}, *S*_2_ = {C, G} would be a valid decomposition for *k* = 1). In this notation, Equation (3) can be rewritten as follows:

(4) $$\;r\left(x,y\right)=\;N\left(x,y\right)\sum_{j=1}^{m}r_{Sj}\left(x,y\right)\;$$

where *m* is the number of different sets, and:

(5) $$\;r_{Sj}\left(x,y\right)=\;\underset{S_{j}}{\sum }\left(x_{\in S_{j}}-4^{-k}\right)\left(y_{\in S_{j}}-4^{-k}\right)\;\;$$

is the part of the correlation coefficient corresponding to the set of DNA words, *S_j_*. The sum over *S_j_* in Equation (5) is defined as the sum over all components of the vectors *x* and *y,* of which indices represent *k*-mer words within the word set *S_j_*. We normalized the results afterwards in the sense that the sum over all respective *r_Sj_* gives 1 (Equation (6)) to make the contributions comparable between regions with different overall correlation values.

(6) $$\;{\hat{r}}_{Sj}\left(x,y\right)=\;\frac{\sum_{S_{j}}\left(x_{\in S_{j}}-4^{-k}\right)\left(y_{\in S_{j}}-4^{-k}\right)\;}{r\left(x,y\right)}\;$$

When we refer to the contribution to the correlation value of a given set, we are always referring to this normalized coefficient ${\hat{r}}_{Sj}\left(x,y\right)$. Since this, again, is a method to calculate pairwise correlations, we use mean values and uncertainties in the same way as we do for complete correlation values (described in Section 2.2).

### 2.4. Software

While other *k*-mer tools, such as JellyFish [20] could have been used to extract the *k*-mer spectra, we decided to use Python and C++ scripts written by the authors to maintain full control of interfaces and workflow of our data structures. We made use of Biopython [21] to handle input files in GenBank and FASTA format. We used the well-known matplotlib library for Python [22] to create the images shown within this article.

All codes and scrips (including visualization) used in this article, as well as a rudimentary English manual, are freely available online at http://www.kip.uni-heidelberg.de/biophysik/software (Oligo Searcher v2.0).

## 3. Results

**Figure 1:**
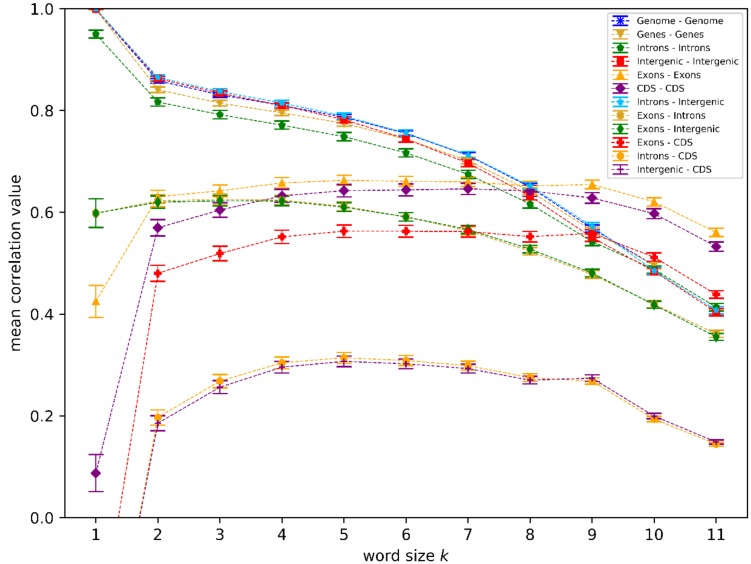
Mean correlation values over all organsims against word length *k*. Shown are the mean values and uncertainties (see Section 2.1 for details) for *k*-mer analysis correlation results between pairs of genome regions. We limited the *y*-axis to values above zero for better readability, and accordingly have not shown the *k* = 1 correlations for Exon–CDS, Introns–CDS, and Intergenic–CDS.

**Figure 2:**
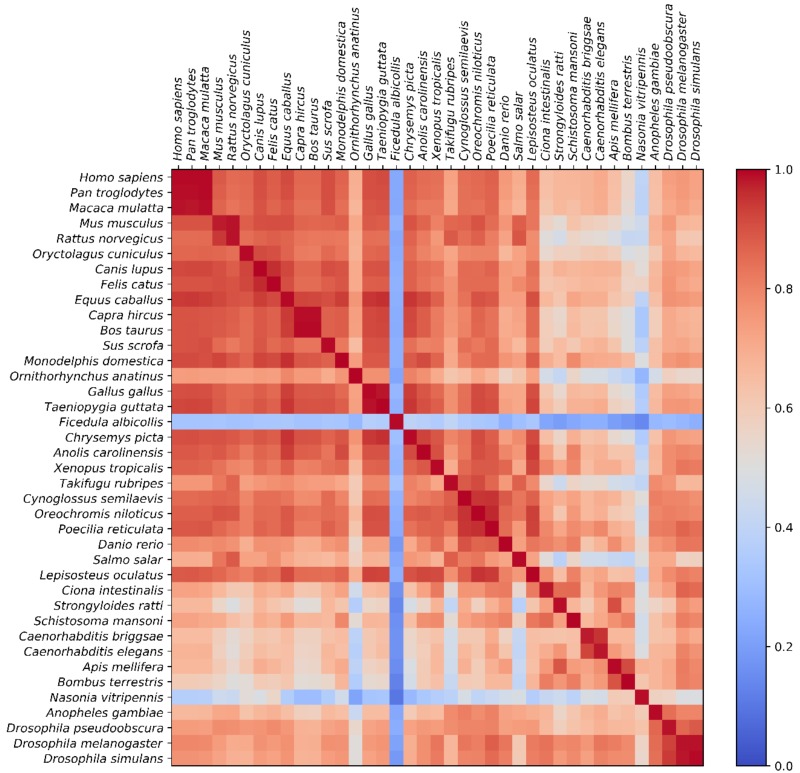
Heatmap of correlations between individual intron and intergenic regions (*k* = 7). Each row represents the pairwise correlation values (Pearson correlation) of introns for the listed organism, while every column is associated with the intergenic region of an organism. Color scale is limited in the heatmap to values above zero for better readability.

We analyzed different genome sequence regions (see Section 2.2) within all 39 organisms mentioned in Table 1 using the *k*-mer analysis described in Section 2.1. We used different word lengths *k* (1 ≤ *k* ≤ 11), and the results are shown in Figure 1. An example of the underlying data for each data point within Figure 1 is shown as a heatmap image in Figure 2 (for *k* = 7, *k* = 11 is given in the Supplementary Materials).

Patterns associated with relatively low correlation values (below 0.4) are visible for *Nasonia vitripennis* and *Ficedula albicollis* in Figure 2. Possible explanations range from special sequence features present within these two species, not conserved within close relatives (e.g., other birds in the case of *Ficedula albicollis*) to quality issues of the two assembled genomes (e.g., within tandem repeats). Since, to our current knowledge, no related discoveries were published, we decided to keep them within our dataset in order to reduce selection bias.

Some of the high correlation values we found can be trivially explained. For small *k* (*k* < 8), the full genome sequences (genome) showed high correlations with intergenic regions since they make up most of the genome sequences. The same is true for correlations between genes and introns, where introns make up most of the gene sequences. It is also not very surprising to see that there is a lower correlation (<0.65) between introns–intergenic regions and exons, and even lower correlation values (< 0.3) between introns/intergenic and CDS (Figure 1). These low correlations between non-coding regions and exons/CDS were expected, since it is known that exons, and especially the CDS, have a well-known conserved biological function (coding for proteins), and thus have associated conserved sequence structures (amino acid codons) [1] whereas known functions and associated sequence structures of introns or intergenic (non-coding) regions are different [23] (e.g., not limited to DNA triplets).

The high correlation values between introns and intergenic regions for small *k* (*k* < 8), visible in Figure 1 and Figure 2 (and in Figure S1 also for *k* = 11, though mainly in the same organism, not between several organisms) and the resulting correlation between the intergenic regions and genes cannot be trivially explained. The correlation between introns and intergenic regions visible in Figure 1 must be the source of this gene–intergenic correlation since there is no significant correlation between exons and intergenic regions. The correlation values, shown as a heatmap in Figure 2, are an illustration of the high correlation between introns and intergenic regions for *k* = 7 (also visible as one respective data point in Figure 1). The correlation values in Figure 2 are relatively high (mostly > 0.6, and visible as mostly red colors in Figure 2) for most pairs of introns and intergenic regions, and especially high if they are in the same organism, as can be seen by the dark red and non-trivial diagonal line. While this feature can also be seen to be a high mean correlation for the respective data point within Figure 1, Figure 2 illustrates that this mean value is not the result of a small number of very high correlations between the genome sequence regions of things such as closely related organisms, but the result of an overall high correlation between regions of all analyzed genome sequence regions. However, as a good illustration of that circumstance, we decided to rely on calculated uncertainties based on standard deviations (shown as error bars within Figure 1) herein. These uncertainties can be interpreted as a summary of the range or spread of values within images like Figure 2. Another interesting feature in Figure 1 is the fact that the correlation values within the exon and CDS regions, as well as the correlation between the two, is significantly lower for small *k* (*k* < 8) when compared to intergenic–intron regions. Since exons and CDS are responsible for important biological functions (coding for proteins), one would expect a very high conservation of sequence structures, and thus relatively high correlation values in relation to non-coding parts like introns or intergenic regions. This behavior is only visible for higher word lengths (*k* > 8) in Figure 1, indicating that these conserved structures found in exons/CDS have a minimum size of about 9 bp on average. This observation is reasonable since proteins typically consist of more than three amino acids (corresponding to three codons, therefore a length of 9 bp of CDS).

The observation that introns and intergenic regions show significantly higher correlation values in Figure 1 for smaller word lengths (*k* < 8) than exons and/or CDS indicates that there are conserved sequence structures with typical length scales of less than 8 bp. To analyze both of these word length regimes, we focused our further analysis on two word lengths for *k* = 7 in the regime of higher non-coding correlations, while still maintaining the optimal *k*-mer word length range for sequences of the size analyzed [16] and *k* = 11, in the regime of *k* > 8 where coding sequences showed higher correlation values. We chose the prime number *k* = 11 over other possibilities like *k* = 9 or *k* = 10 to exclude resonance effects, which are potentially caused by smaller structures, such as amino acid codons (e.g., because 9 is a multiple of 3). This also further motivated the usage of *k* = 7 over other values like *k* = 6.

We analyzed the *k*-mer words responsible for interesting correlations at *k* = 7 and *k* = 11 by applying a decomposition of the correlation values as described in Section 2.3. The word sets we used consisted of one word of respective length *k* within each set (e.g., for *k* = 7: *S*_1_ = {AAAAAAA}, *S*_2_ = {AAAAAAC}, *S*_3_ = {AAAAAAG}, *S*_4_ = {AAAAAAT}, *S*_5_ = {AAAAACA}, …). This decomposition obviously satisfies the requirements of a valid decomposition described in Section 2.3. The *k*-mer words which contributed most to high correlations (Figure 1) are shown in Table 3 (*k* = 7) and Table 4 (*k* = 11).

**Figure 3:**
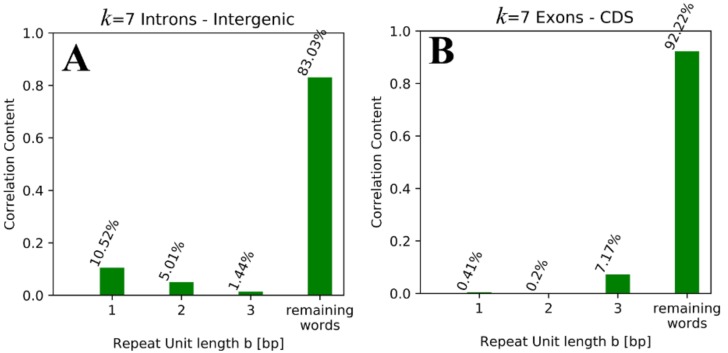
Histograms representing the correlation contributions of tandem repeat *k*-mer words with different repeat unit lengths to *k* = 7 correlation values for intron–intergenic correlation (**A**) and to exon–CDS correlation (**B**) (see Section 2.2 for details).

The top 10 words shown in Table 3 and Table 4 show clear preferences of specific *k*-mer word patterns for different pairs of regions compared. Even though only the top 10 words are shown, the tandem repeats observed also dominate in complete lists (not shown for reasons of clarity; for the top 100 see Supplementary Materials, Table S2 for *k* = 7, and Table S3 for *k* = 11). The most obvious feature shared between all pairs of regions shown in Table 3 and Table 4 is the fact that tandem repeats (e.g., multiple repetitions of one small unit, such as ATATATA, is a tandem repetition of the repeat unit AT), with repeat unit lengths smaller than 4, make up a considerable fraction of the top 10 contributing words between all pairs of sequences (36–100%) (Figure 3). If the contribution was to be evenly distributed over all *k*-mer words, the correlation value should be about 4^−*k*^; therefore one would expect contributions to be lower than 0.0006 for *k* = 7 and even less for *k* = 11. Since there are only 4^b^ possible tandem repeats for a given length b (in base pairs) of the repeat unit, one would expect a fraction of 4^b^/4^k^ = 4^b−*k*^ (~0.4% for b = 3 and *k* = 7, and even less for b < 3 or *k* = 11) for tandem repeat *k*-mer words within the top 10 contributing words. Especially when small deviations in the sequences between the listed words and tandem repeats (e.g., one mismatch) are considered, nearly 100% of the words shown are tandem repeat-based. Accordingly, tandem repeats seem to be overrepresented within the lists shown in Table 3 and Table 4, in which the overrepresented repeat unit length also differs depending on the pair of regions compared. Lists of top 10 (and also top 100) contributing words pairing CDS are dominated by unit lengths of 3 bp. This is partially true also for pairing exons or even CDS and exons, especially for lower *k* (*k* = 7). For other pairs, the lists contain mainly tandem repeats with smaller unit lengths of 1 bp and 2 bp (partially true also for exons, especially with higher *k*, *k* = 11). The lists shown in Table 3 and Table 4 also show that except for pairs containing CDS, the top contributing words are A/T words (*k*-mer words consisting of A and T only), while C/G words are only rarely found. And even though polyA and polyT words give a large contribution to the correlation value, the contribution of all tandem repeats with *b* = 2 together is bigger.

The data shown in Table 3 and Table 4 clearly suggest that the structures of most-contributing *k*-mer words are different for the intron–intergenic correlation when compared with the exons–CDS, even though exons seem to be a hybrid under this perspective between CDS and intron–intergenic regions. This indicates a different nature of underlying sequence structures responsible for high correlations found by the applied *k*-mer analysis. It is peculiar that the top-contributing words found for CDS, and to some extent also for exons, represent tandem repeat patterns of amino acid codons, namely units of 3 bp (e.g., (ATT)_n_), while the words found for intron–intergenic regions show different patterns.

To further quantify these results, we went from analyzing structures of the top contributing words to analyzing the contributions of all words representing tandem repeats, with a given repeat unit length b. In the context of correlation contribution, this could be achieved by defining the following four different word sets: *S*_1_ = {(X)_n_ for X ∈ {A,C,G,T}}, *S*_2_ = {(XY)_n_ for X, Y ∈ {A,C,G,T} and not in *S*_1_}, *S*_3_ = {(XYZ)_n_ for X, Y, Z ∈ {A,C,G,T} or *S*_1_ or *S*_2_}, *S*_4_ = {not element of *S*_1_, *S*_2_, *S*_3_}. One can easily check that this decomposition satisfies the requirements of a valid decomposition, since the sets are all mutual exclusives with *S*_1_–*S*_3_ containing tandem repeats with repeat unit lengths of 1–3, respectively, and *S*_4_ contains all remaining *k*-mer words (including all non-tandem repeat words). Using these sets, we performed a decomposition of the correlation coefficient, as described in Section 2.2. The exemplary results, which show contributions of different repeat unit lengths *b*, are shown in Figure 3 and listed for all interesting pairs in Table 5.

Table 5. While non-tandem repeat words contribute more than 80% (for *k* = 7) to the correlation values of all pairs, tandem repeats with unit lengths of 1 bp and 2 bp contribute significantly to the correlations within and between introns and intergenic regions, while repeats with unit lengths of 3 bp contribute mostly to correlations within and between exons and CDS. Once again, exons also show some features of introns and intergenic regions, as for exons–exons, the amount of unit lengths of 1 bp is also quite high. The reason behind this observation could arguably be the known presence of poly-A tracts within UTRs, which are part of exons but not part of the CDS [24]. Table 3 shows that the highest-contributing words between exons and introns are mainly Poly-A/T words, which supports this interpretation.

For *k* = 11, the contributions of tandem repeats further increase to more than 50% for pairs of introns and intergenic regions. This supports the observation made in Table 3 and Table 4, as well as the idea that the repeat unit length for exons and CDS is associated with conserved sequence structures (amino acid codons), while the sequence structures conserved between introns and intergenic regions consist of smaller repeat units. It is remarkable that the remaining words contribute more to the correlation values of exons (> 72%) and CDS (> 89%), while values < 85% (down to < 50%) are observed for introns and intergenic regions.

The fact that more than 80% of contributions are based on non-tandem repeat words for *k* = 7 could lead to the misleading impression that tandem repeat words are proven to be irrelevant for the observed correlations and therefore for conserved sequence structures, but one should keep in mind that for *k* = 7 there are only 4^3^ = 64 tandem repeat words with unit length b < 4, while there are 4^7^ = 16,384 words overall, meaning that ~0.4% of all *k*-mer words are responsible for 8–18% (depending on the paired regions) of the respective correlation values.

Besides the fact that tandem repeat words are overrepresented within the top-contributing word lists, shown in Table 3 and Table 4, it is also clear that there is a tendency for A/T-rich words in the top-contributing words to be in intron intergenic regions, and an opposite tendency for top-contributing words between CDS and exons. To quantify this observation, we performed a correlation decomposition based on the G/C content of respective *k*-mer words by defining sets like *S*_G/C<X%_ = {words with G/C content < X%} (*S*_A/T<X%_ can be defined analogously). The results, shown in Table 6, supported the observations concerning the G/C (or A/T content) made in Table 3 and Table 4. 

Words with low G/C content contribute most to the correlation values between intron and intergenic regions while words with a relatively balanced G/C to A/T ratio contribute most to the correlation values between exons and CDS for *k* = 7 and *k* = 11. This could illustrate constraints to the sequence structures of CDS and exons, resulting from the need to code for many different amino acids, in the sense that there would not be enough words consisting of mainly A/T or mainly G/C to encode for all amino acids with a reasonable level of redundancy. The fact that the G/C content in the words contributing most to the correlation values between introns and intergenic regions is very low, while words with high amounts of G/C contribute very little to the correlations between introns and intergenic regions, supports the idea that the underlying conserved sequence structures do not encode for biological functions in the same way as CDS does.

We further investigated the preference for low G/C or A/T rich words respectively, within introns and intergenic regions by analyzing tandem repeat *k*-mer words allowing one mismatch (e.g., AAAAGAA is counted as AAAAAAA). That corresponding words contribute significantly to the correlation between introns and intergenic regions is illustrated by the abundances of these words within the top 10 contributing word lists shown in Table 3 and Table 4 (or top 100 in Supplementary Materials, Table S2 and Table S3). Table 7 shows the contribution of all tandem repeat words with unit lengths *b* < 3 to the correlation between introns and intergenic regions for *k* = 7 and *k* = 11. The decomposition was made analogous to the decomposition for creating the top lists in Table 3 by allowing one mismatch for tandem repeat identification.

Table 7 again shows that words contribute more to the correlation between introns and intergenic regions if they have lower G/C content for *k* = 7 and *k* = 11. The contribution of words consisting of A/T only reaches up to 10%, while words consisting of G/C only contribute down to 0.01% for (GC)_n_ and (CG)_n_. The contributions of the complementary DNA words (AC)_n_, (CA)_n_ and (TG)_n_, (GT)_n_ show slightly higher contributions than other words with the same G/C content for *k* = 7, and this discrepancy to the general trend further increases for *k* = 11. The high contribution of non-polyA and non-polyT words taken together is also noteworthy.

To check possible indications on whether these words discovered were completely without function (a product of random mutations) or whether they lay in an unfunctional region, we analyzed possible preferences for specific nucleotides (A, C, G, T) within mismatches for all tandem repeat *k*-mer words with unit lengths < 3 bp, allowing only one mismatch. The results within introns and intergenic regions are shown in Table 8. Assuming that tandem repeats with mismatches were, to a significant extent, the results of point mutations in previously complete repeat words or sequence patterns mutating towards tandem repeats, these results can be interpreted as being preferences for mutations nearby or within tandem repeat words. Similar methods were developed, published, and used in recent publications [7,18]. While further investigation would be needed for a more definitive statement, the observation that these mismatches do not seem to be equivalently distributed for all four bases or all words could indicate that these sequences are functional.

While the general tendency towards mismatches consisting of A or T could be explained by a higher A/T content in general within analyzed organisms (data not shown), the fact that there are differences in mismatch preferences between different *k*-mer words cannot be explained by global A/T or G/C content. The same is true for both introns and intergenic regions, as these preferences are conserved between them. Table 8 shows that for *k* = 7, the mismatches preferred are A/T over G/C. This does not occur randomly, as A is preferred when already containing A, and T if already containing T, and this tendency increases with the content of A/T (Poly-A and Poly-T are excluded since they cannot have mismatches of A and T respectively). If interpreted as point mutations, this indicates that there is a tendency of mutation towards higher A content in words containing A and towards higher T content in words containing T. An analogous tendency for C or G was not found (see Table 8). This further supports the idea that sequence structures containing high content of A or T are preferred. The general tendency also exists for *k* = 11, with the exception that (AC)_n_, (CA)_n_, (TG)_n_, and (GT)_n_ do not show this tendency for *k* = 11. They even show a slight tendency toward the complementary nucleotide (i.e., more T mismatches in words containing A and more A mismatches in words containing T). It is remarkable that these belong in the set of words that showed increased correlation contributions for *k* = 7 and especially for *k* = 11 (see Table 7), apart from pure A/T words. If interpreted as mutations, this could indicate that repeats of (AC)_n_, (CA)_n_, (TG)_n_, and (GT)_n_ do not mutate into homogeneous poly-A/T sequences. That these observations concerning (AC)_n_, (CA)_n_, (TG)_n_, and (GT)_n_, and other *k*-mer words are conserved between introns and intergenic regions in Animalia supports the idea of a function shared between introns and intergenic regions.

## 4. Discussion and Conclusions

In summary, conserved sequence structures between different genomic regions were found, with positive correlations between sequence regions already known to be highly conserved (e.g., exons and CDS), as well as between regions where conservations could not be trivially explained as being in and between introns and intergenic regions. The general observation that short nucleotide units, such as dinucleotides and trinucleotides, are most relevant for sequence structures is consistent with the discoveries of former studies [6].

All correlations within exons and CDS and between the two were the result of tandem repeats, with repeat unit lengths of 3 bp (triplet repeats). This observation is consistent with the well-known sequence patterns (amino acid codons) which are responsible for their biological function (coding for proteins) and accordingly responsible for their conservation [1]. While the amino acid code alone cannot explain the relevance of repeats, some amino acid codon triplet repeats, consistent with the *k*-mer words identified as being most relevant for exon/CDS correlations (as shown in Table 3 and Table 4), are known to code for important protein structures [25]. The observation that for *k* = 7 and *k* = 11 non-repeat words still contribute more than 70% to the correlation values of exons and CDS (as shown in Table 5A,B), as well as the relevance of words with balanced G/C to A/T content most contributing to exon and CDS correlations (as shown in Table 6A,B), support the argument that the correlation of the two regions is a product of sequence conservation due to constraints based on the included amino acid code, since these observations can be explained by the need of many different DNA words as (redundant) codons for amino acids. The presence of smaller repeat units and the slightly higher relevance of A/T words for exon–exon correlations, when compared to correlations with CDS as well as other described similarities between exons on one side and introns and intergenic regions on the other, show the hybrid composition of exons. This can be explained by the presence of UTR which are part of exons but not of CDS. Accordingly, sequence structures in exons deviating from the structures found in CDS (as observed within this article) are expectable because UTRs do not code for amino acids and thus are not affected by amino acid codon-related constraints. In summary, the correlation found between exons and CDS, as well as the responsible sequence structures identified in this article, are consistent with known conserved sequence properties of these genome regions. 

The correlation between non-coding regions (introns and intergenic regions) cannot be explained as easily. The difference between structures identified as being responsible for correlations of non-coding regions compared to coding regions (e.g., exons, CDS) is consistent with the fact that they do not share the same function (coding for proteins or functional RNA). The most impressive results are the high conservation of tandem repeats with a repeat unit length of *b* ≤ 2 bp between introns and intergenic regions in each of the analyzed organisms, as well as the high conservation between these regions when compared across the species, mainly in Deuterostomia. The observation that introns and intergenic regions show high correlations and the same sequence patterns responsible for intron—intron and intergenic—intergenic correlations are also responsible for intron—intergenic correlations indicates phylogenetic conservation of these structures. As this is commonly seen as a sign for preserved function, the possibility of functional elements shared between introns and intergenic regions as an explanation for the observations made in this article, seems to be the most promising hypothesis. The identification of tandem repeats with b ≤ 2 bp favors this, as the set of words found is stable over large parts of evolution, for the big group of Deuterostomia at least for 500 million years ago, despite all base mutations, insertions, deletions, or rearrangements which have occurred during all these ages. And also, despite all these evolutionary alterations, correlation values of *k*-mer distributions in intron and intergenic regions, strongly being under domination of these tandem repeats, are, in each organism, very high (above 0.8), higher than between species, indicating a highly preserved but not fixed homogeneity. This idea gets even higher support by the analysis of single-base mismatches in these DNA words, as these regions do not seem, for the exception of pure C/G words, to preserve every possible observable mutation, but favoring, depending on their own sequence, special transitions, mainly towards A/T. It may be necessary here to say that even though polyA and polyT have a huge influence in all analyses, the obsesrved patterns and properties are more strongly influenced by the sum of all other tandem repeats with *b* ≤ 2 bp and may not be explained by just arguing on polyA and polyT. How these preservations are maintained or where they may have risen from remains debatable. One explanation for conserved structures between introns and intergenic regions could be a shared origin of, or a constant exchange between both regions. A corresponding observation (emergence of a gene from intergenic DNA in *Mus musculus*) was made in [26]. In this case, it could be beneficial to conserve sequence structures between intergenic regions as a source and introns as a target, to reduce the number of mutations needed for an active gene to emerge. While this hypothesis could explain the observed correlations between introns and intergenic regions, our observations do not support or refute it. The reason for this is that conserved sequence structures could well be a disadvantage, since a small number of mutational steps needed to create genes from intergenic regions may also lead to many potentially harmful active genes. 

Nevertheless, for the large sequence amounts of introns and intergenic regions, this rarely-observed event is not so convincing. The opposite pathway, namely, intergenic regions as products of gene (mainly consisting of introns) degeneration, supported by the existence of pseudogenes still recognizable to have gene-like sequence structures but not coding for proteins [27], is also possible and was, in fact, one of the first theories about the origin of non-coding DNA [3,28]. This mechanism can only explain conserved sequence structures, if intergenic regions already include functional DNA motifs that are then shared between introns and intergenic regions or do not have enough time to deviate from intron-like sequence structures through mutations. The last possibility seems unreasonable considering the time for mutations corresponding to the phylogenetic range of organisms analyzed (about 500–600 million years), especially since tandem repeats in general are known to have high mutational rates [29]. 

A possible family of functional DNA motifs known to be shared between introns and intergenic regions are transposons. Since these functions were found to be associated with embryogenesis [30], a special role of transposons for sequence-structure conservation within Animalia would not be surprising. Transposons make up a considerable amount of genome sequences of Animalia and accordingly intergenic regions. Retrotransposons especially are found in very large numbers [31] in many Animalia species. Since retrotransposons are found as repetitions of similar sequence motifs within genome sequences, they could, in general, explain conserved sequence structures as observed. While a quantitative determination of the roles of transposons for the correlation content would require a formal analysis of sequence patterns of all known transposon families in Animalia (which is out of the scope of this article), the first qualitative results suggest that the tandem repeat structures we found to be responsible for high correlation values are not typical for sequence patterns of transposons [31,32]. Transposons are also known to be flanked by direct repeats, but these repetitions are usually not tandem repeats. An exception to this is Poly-A, found as (A/T)_7_ and (A/T)_11_ in this article. Poly-A (Poly-T on the opposing strand) tracks are known to flank many transposons as a result of reverse transcription [24]. They could, in principle, explain the occurrence of A_n_ and T_n_ words within our analysis, but not the presence of other tandem repeats. 

Another known functional sequence motif conserved between introns and intergenic regions, present in all analyzed organisms, are transcriptional factors (more precisely, their binding sites), especially binding motifs for silencers and enhancers [33]. While the exact sequence structure of these transcriptional-factor binding sites is not completely understood [23], and while a formal analysis would again be required to confirm these qualitative results, patterns typically annotated as such binding sites do not show a significant amount of tandem repeats [34]. Like transposons, transcriptional-factor binding sites were found to be flanked by sequences consisting of either A or T, but since their typical size is 3 bp–5 bp [34], they cannot be responsible for the results we found with word lengths > 6 bp.

Another discovery made is that transcriptional factors are associated with local DNA topography [36]. This observation is of special interest for the interpretation of the observations made in this article, since dinucleotides (DNA words of length 2 bp) are often used to determine such structural and topological properties of DNA molecules [36,37] and the structures we observed to contribute most to intron intergenic correlations consist mainly of repeats of such dinucleotides. Since transcriptional factors influence distant genes through DNA loops [33], it seems reasonable that local physical DNA properties that favor such loops are beneficial when associated with transcriptional factor binding sites. Since A/T-rich sequence structures—found responsible for introns/intergenic correlation in this article—were observed to induce loops within DNA molecules [38], this is suggestive of the hypothesis that the function potentially associated with conserved sequence structures between introns and intergenic regions could be associated with local bending properties, and thus with 3D or more general conformation, with the topology of the DNA molecules. If true, the role of the different observations of (AC)_n_, (CA)_n_, (TG)_n_, and (GT)_n_ in contrast to (AG)_n_, (GA)_n_, (TC)_n_, and (CT)_n_ could be associated with specific structural dimer properties. The tandem repeat sequences identified in this article could also influence transcriptional-factor activity, and likewise, DNA topology through their mere presence, since the distance between transcriptional-factor and regulated genes would be increased or decreased based on the length of the tandem repeats located between them, and the same is true for the general length of the DNA molecules which could influence their general topological properties (sequence-dependent and sequence-independent).

If this is true, the fast mutational rates of tandem repeats and their tendency to change rapidly in length [29], manuscript in preparation, could favor them over other sequence patterns to allow relatively fast adaptions of this regulatory function. In this case, the explanation of the preference of specific tandem repeats over others, especially the observed preference of A/T-rich repeats in these assumed interactions, may be an important and promising stepping-stone for further research in this field.

## Figures and Tables

**Table 1:** Organisms and chromosomes used in our analysis (accession numbers of sequences are found within the appendix, Table S1).

Species	Further Classification	Chromosomes
*Homo sapiens*	Deuterostomia—Mammalia	1–22, X, Y
*Pan troglodytes*	Deuterostomia—Mammalia	1, 2A, 2B, 3-22, X, Y
*Mus musculus*	Deuterostomia—Mammalia	1–19, X, Y
*Rattus norvegicus*	Deuterostomia—Mammalia	1–20, X, Y
*Oryctolagus cuniculus*	Deuterostomia—Mammalia	1–21, X
*Canis lupus*	Deuterostomia—Mammalia	1–38, X
*Felis catus*	Deuterostomia—Mammalia	A1–A3, B1–B4, C1, C2, D1–D4, E1–E3, F1, F2, X
*Equus caballus*	Deuterostomia—Mammalia	1–31, X
*Capra hircus*	Deuterostomia—Mammalia	1–29
*Bos taurus*	Deuterostomia—Mammalia	1–29, X
*Sus scrofa*	Deuterostomia—Mammalia	1–18, X, Y
*Monodelphis domestica*	Deuterostomia—Mammalia	1–8, X
*Ornithorhynchus anaticus*	Deuterostomia—Mammalia	1–7, 10–12, 14, 15, 17, 18, 20, X1, X2, X3, X5
*Gallus gallus*	Deuterostomia—Aves	1–28, 30, 33, W, Z
*Taeniopygia guttata*	Deuterostomia—Aves	1, 1A, 1B, 2-4, 4A, 5–15, 17–28, Z
*Ficedula albicollis*	Deuterostomia—Aves	1–15, 17–28, 1A, 4A, Z
*Chrysemys picta*	Deuterostomia—Reptilia	1–11, 13, 15, 17, 19, 21, 22, 24, 25
*Anolis carolinensis*	Deuterostomia—Reptilia	1–6, a, b, c, d, f, g, h
*Xenopus tropicalis*	Deuterostomia—Amphibia	1–10
*Takifugu rubripes*	Deuterostomia—Osteichthyes	1–22
*Cynoglossus semilaevis*	Deuterostomia—Osteichthyes	1–20, W, Z
*Oreochromis niloticus*	Deuterostomia—Osteichthyes	1, 2, 3a, 3b, 4–22
*Poecilia reticulata*	Deuterostomia—Osteichthyes	1–23
*Danio rerio*	Deuterostomia—Osteichthyes	1–25
*Salmo salar*	Deuterostomia—Osteichthyes	1–29
*Lepisosteus oculatus*	Deuterostomia—Osteichthyes	1–29
*Ciona intestinalis*	Deuterostomia—Ascidiae	1–14
*Schistosoma mansoni*	Protostomia—Trematoda	1–7, W
*Strongyloides ratti*	Protostomia—Secernentea	1, 2, X
*Caenorhabditis brigsae*	Protostomia—Secernentea	1–5, X
*Caenorhabditis elegans*	Protostomia—Secernentea	1–5, X
*Apis mellifera*	Protostomia—Insecta	1–16
*Bombus terrestris*	Protostomia—Insecta	1–18
*Nasonia vitripennis*	Protostomia—Insecta	1–5
*Anopheles gambiae*	Protostomia—Insecta	2L, 3L, 2R, 3R, X
*Drosphila pseudoobscura*	Protostomia—Insecta	2, 3
*Drosophila melanogaster*	Protostomia—Insecta	2L, 2R, 3L, 3R, 4, X, Y
*Drosohpila simulans*	Protostomia—Insecta	2L, 3L, 2R, 3R, 4, X,

**Table 2:** Definitions of genome sequence regions.

Region ID	Definition used for the Algorithm	Definition/Description
Genome	Complete genome sequence	Complete genome sequence of the respective organism.
Genes	Genes *	Genes of a respective organism (including pseudo and RNA genes **).
Exons	Exons *	Exons (transcribed parts) of genes
		(including pseudo and RNA genes).
Introns	Genes * with exons * masked	Introns (untranscribed parts) of genes
		(including pseudo and RNA genes).
CDS	Coding DNA sequences (CDS *)	CDS (translated parts) of exons
		(exons without untranslated regions (UTRs)).
Intergenic	Complete genome sequence with genes (and centromeres) masked.	Non-coding regions between genes (RNA genes are also considered genes in this context).

*: Referring to the labels found in respective GenBank files [14]. **: RNA genes including tRNA, ncRNA (e.g., long ncRNA, micro RNA, snoRNA).

**Table 3:** List of 10 *k*-mer words with highest contribution to correlation values for *k* = 7. Organism Content, shown for homogenous pairings of sequence regions, is defined by the fraction of analyzed organisms for which the difference between content of the corresponding *k*-mer word and the mean *k*-mer word content (4^−*k*^) is larger than 1 σ with respect to the *k*-mer word content distribution of the respective organism.

Correlated Regions	*k*-mer Word	Contribution	Organism Content
**Exons**–**Exons**	TTTTTTT	0.0204	100%
	AAAAAAA	0.0196	100%
	GCTGCTG	0.0045	92.3%
	CAGCAGC	0.0044	89.7%
	CTGCTGC	0.0032	82.1%
	GCAGCAG	0.0032	84.6%
	TTTTCTT	0.0029	100%
	TTTCTTT	0.0028	100%
	TTTATTT	0.0028	97.4%
	TGCTGCT	0.0027	92.3%
**CDS**–**CDS**	GCTGCTG	0.0077	94.9%
	CAGCAGC	0.0077	94.9%
	CTGCTGC	0.0054	92.3%
	GCAGCAG	0.0053	89.7%
	TGCTGCT	0.0042	94.9%
	AGCAGCA	0.0041	94.9%
	CTCCTCC	0.0035	89.7%
	GGAGGAG	0.0035	89.7%
	CTTCTTC	0.0031	100%
	GAAGAAG	0.0030	100%
**Introns**–**Introns**	TTTTTTT	0.0553	100%
	AAAAAAA	0.0552	100%
	TGTGTGT	0.0070	89.7%
	ACACACA	0.0070	87.2%
	TTTATTT	0.0065	100%
	AAATAAA	0.0064	100%
	ATATATA	0.0063	100%
	TATATAT	0.0063	100%
	ATTTTTT	0.0056	100%
	GTGTGTG	0.0056	82.1%
**Intergenic**–**Intergenic**	TTTTTTT	0.0467	100%
	AAAAAAA	0.0466	100%
	ATATATA	0.0073	100%
	TATATAT	0.0072	100%
	AAATAAA	0.0069	100%
	TTTATTT	0.0069	100%
	TGTGTGT	0.0059	89.7%
	ACACACA	0.0057	89.7%
	ATTTTTT	0.0055	100%
	AAAAAAT	0.0055	100%
**Introns**–**Intergenic**	TTTTTTT	0.0510	
	AAAAAAA	0.0510	
	ATATATA	0.0068	
	TATATAT	0.0068	
	AAATAAA	0.0066	
	TTTATTT	0.0066	
	TGTGTGT	0.0064	
	ACACACA	0.0063	
	ATTTTTT	0.0056	
	AAAAAAT	0.0056	
**Exons**–**Introns**	TTTTTTT	0.0414	
	AAAAAAA	0.0406	
	TTTATTT	0.0052	
	AAATAAA	0.0047	
	ATTTTTT	0.0044	
	TTTAAAA	0.0042	
	TTTTAAA	0.0042	
	AAAAAAT	0.0041	
	ACACACA	0.0040	
	TGTGTGT	0.0039	
**Exons**–**CDS**	GCTGCTG	0.0065	
	CAGCAGC	0.0065	
	CTGCTGC	0.0047	
	GCAGCAG	0.0046	
	TGCTGCT	0.0037	
	AGCAGCA	0.0037	
	GGAGGAG	0.0030	
	CTCCTCC	0.0030	
	TTCTTCT	0.0025	
	AGAAGAA	0.0025	

**Table 4:** List of 10 *k*-mer words with highest contribution to correlation values for *k* = 11. Organism Content, shown for homogenous pairings of sequence regions, is defined by the fraction of analyzed organisms for which the difference between content of the corresponding *k*-mer word and the mean *k*-mer word content (4^−*k*^) is larger than 1 σ, with respect to the *k*-mer word content distribution of the respective organism.

Correlated Regions	*k*-mer word	Contribution	Organism Content
**Exons**–**Exons**	TTTTTTTTTTT	0.0662	100%
	AAAAAAAAAAA	0.0657	100%
	ACACACACACA	0.0155	97.4%
	CACACACACAC	0.0148	97.4%
	TGTGTGTGTGT	0.0148	92.3%
	GTGTGTGTGTG	0.0142	94.9%
	ATATATATATA	0.0069	97.4%
	TATATATATAT	0.0068	97.4%
	TGCTGCTGCTG	0.0052	100%
	CAGCAGCAGCA	0.0052	100%
**CDS**–**CDS**	TGCTGCTGCTG	0.0106	100%
	CAGCAGCAGCA	0.0101	100%
	GCTGCTGCTGC	0.0082	100%
	CTGCTGCTGCT	0.0079	100%
	GCAGCAGCAGC	0.0078	100%
	AGCAGCAGCAG	0.0075	100%
	TCCTCCTCCTC	0.0051	100%
	GAGGAGGAGGA	0.0050	100%
	CCTCCTCCTCC	0.0036	100%
	GGAGGAGGAGG	0.0035	100%
**Introns**–**Introns**	AAAAAAAAAAA	0.0990	100%
	TTTTTTTTTTT	0.0987	100%
	TGTGTGTGTGT	0.0480	97.4%
	ACACACACACA	0.0478	97.4%
	GTGTGTGTGTG	0.0455	97.4%
	CACACACACAC	0.0453	97.4%
	ATATATATATA	0.0363	100%
	TATATATATAT	0.0363	100%
	AGAGAGAGAGA	0.0167	97.4%
	TCTCTCTCTCT	0.0165	100%
**Intergenic**–**Intergenic**	TTTTTTTTTTT	0.0837	100%
	AAAAAAAAAAA	0.0835	100%
	TGTGTGTGTGT	0.0452	97.4%
	ACACACACACA	0.0440	97.4%
	GTGTGTGTGTG	0.0428	97.4%
	TATATATATAT	0.0422	100%
	ATATATATATA	0.0422	100%
	CACACACACAC	0.0417	97.4%
	AGAGAGAGAGA	0.0193	100%
	TCTCTCTCTCT	0.0192	100%
**Introns**–**Intergenic**	AAAAAAAAAAA	0.0884	
	TTTTTTTTTTT	0.0883	
	TGTGTGTGTGT	0.0456	
	ACACACACACA	0.0448	
	GTGTGTGTGTG	0.0432	
	CACACACACAC	0.0425	
	ATATATATATA	0.0395	
	TATATATATAT	0.0395	
	AGAGAGAGAGA	0.0181	
	TCTCTCTCTCT	0.0178	
**Exons**–**Introns**	TTTTTTTTTTT	0.1002	
	AAAAAAAAAAA	0.0999	
	ACACACACACA	0.0344	
	TGTGTGTGTGT	0.0338	
	CACACACACAC	0.0328	
	GTGTGTGTGTG	0.0323	
	ATATATATATA	0.0223	
	TATATATATAT	0.0222	
	AGAGAGAGAGA	0.0096	
	TCTCTCTCTCT	0.0095	
**Exons**–**CDS**	TGCTGCTGCTG	0.0093	
	CAGCAGCAGCA	0.0091	
	GCTGCTGCTGC	0.0073	
	CTGCTGCTGCT	0.0071	
	GCAGCAGCAGC	0.0071	
	AGCAGCAGCAG	0.0069	
	GAGGAGGAGGA	0.0044	
	TCCTCCTCCTC	0.0044	
	CCTCCTCCTCC	0.0032	
	GGAGGAGGAGG	0.0032		

**Table 5:** Correlation contributions of tandem repeats with different repeat unit lengths b (in base pairs) for word lengths k = 7 and k = 11, between different genome regions analogous to the data shown in Figure 2.

Correlated Regions	*k*	Correlation Contribution *b* = 1	Correlation Contribution *b* = 2	Correlation Contribution *b* = 3	Remaining Words
**Exons**—**Exons**	7	4.02%	0.98%	5.18%	89.82%
**CDS**—**CDS**	7	0.03%	0.09%	8.21%	91.67%
**Exons**—**CDS**	7	0.41%	0.20%	7.17%	92.22%
**Exons**—**Introns**	7	8.28%	2.81%	2.26%	86.66%
**Introns**—**Introns**	7	11.22%	5.08%	1.36%	82.34%
**Intergenic**—**Intergenic**	7	9.62%	4.82%	1.54%	84.02%
**Introns**—**Intergenic**	7	10.52%	5.01%	1.44%	83.03%
**Exons**—**Exons**	11	13.37%	8.70%	5.70%	72.23%
**CDS**—**CDS**	11	0.02%	0.03%	9.86%	90.09%
**Exons**—**CDS**	11	0.46%	0.60%	9.30%	89.65%
**Exons—Introns**	11	21.31%	21.49%	2.37%	54.83%
**Introns—Introns**	11	20.45%	32.35%	1.54%	45.66%
**Intergenic—Intergenic**	11	17.34%	33.40%	2.09%	47.27%
**Introns—Intergenic**	11	18.51%	32.47%	1.84%	47.18%

**Table 6:** Correlation contributions of words with different G/C contents for word lengths *k* = 7 and *k* = 11, between different genome regions.

Correlated Regions	*k*	0% G/C	0–25% G/C	25–50% G/C	50–75% G/C	75–100% G/C	100% G/C
**Exons**—**Exons**	7	11.71%	23.53%	34.13%	39.23%	3.11%	0.21%
**CDS**—**CDS**	7	0.38%	4.33%	38.89%	52.92%	3.86%	0.35%
**Exons**—**CDS**	7	1.91%	7.95%	39.16%	49.27%	3.63%	0.29%
**Exons**—**Introns**	7	23.99%	42.64%	28.35	26.60%	2.41%	0.09%
**Introns**—**Introns**	7	33.02%	55.66%	21.94%	18.82%	3.59%	0.61%
**Intergenic**—**Intergenic**	7	32.49%	56.00%	22.01%	18.31%	3.69%	0.71%
**Introns**—**Intergenic**	7	32.83%	55.89%	21.92%	18.49%	3.71%	0.75%
**Exons**—**Exons**	11	18.84%	33.66%	29.21%	33.72%	3.41%	1.06%
**CDS**—**CDS**	11	0.16%	3.56%	34.72%	56.19%	5.53%	1.32%
**Exons**—**CDS**	11	1.24%	8.30%	35.78%	50.63%	5.29%	1.52%
**Exons**—**Introns**	11	32.51%	52.23%	25.27%	20.63%	1.86%	1.33%
**Introns**—**Introns**	11	37.31%	56.78%	23.69%	18.35%	1.18%	0.69%
**Intergenic**—**Intergenic**	11	36.08%	56.25%	24.37%	18.28%	1.11%	0.63%
**Introns**—**Intergenic**	11	36.27%	56.06%	24.08%	18.53%	1.34%	0.84%

**Table 7:** Correlation contributions for correlations between introns and intergenic regions for tandem repeat *k*-mer words with repeat unit length ≤ 2 bp for either no or one allowed mismatches, for *k* = 7 (top) and *k* = 11 (bottom).

*k*-mer Word	No Mismatch	1 Mismatch
AAAAAAA	5.10%	11.51%
CCCCCCC	0.17%	0.20%
GGGGGGG	0.15%	0.18%
TTTTTTT	5.10%	11.55%
ACACACA	0.63%	0.88%
AGAGAGA	0.38%	0.70%
ATATATA	0.68%	1.64%
CACACAC	0.50%	0.57%
CGCGCGC	<0.01%	0.08%
CTCTCTC	0.30%	0.43%
GAGAGAG	0.30%	0.43%
GCGCGCG	<0.01%	0.08%
GTGTGTG	0.51%	0.58%
TATATAT	0.68%	1.63%
TCTCTCT	0.38%	0.70%
TGTGTGT	0.64%	0.89%
AAAAAAAAAAA	8.84%	10.90%
CCCCCCCCCCC	0.42%	0.45%
GGGGGGGGGGG	0.41%	0.44%
TTTTTTTTTTT	8.83%	10.89%
ACACACACACA	4.48%	4.61%
AGAGAGAGAGA	1.81%	1.91%
ATATATATATA	3.95%	4.20%
CACACACACAC	4.25%	4.36%
CGCGCGCGCGC	<0.01%	<0.01
CTCTCTCTCTC	1.68%	1.79%
GAGAGAGAGAG	1.70%	1.79%
GCGCGCGCGCG	<0.01%	<0.01%
GTGTGTGTGTG	4.32%	4.43%
TATATATATAT	3.95%	4.19%
TCTCTCTCTCT	1.78%	1.89%
TGTGTGTGTGT	4.56%	4.68%

**Table 8:** Contents of specific nucleotides (A, C, G, T) within mismatches of tandem repeat *k*-mer words (for *k* = 7 and *k* = 11). Contents lower than 20% are marked red, and contents higher than 30% are marked green, remaining contents are marked yellow.

*k*-mer Word	Introns A	C	G	T	Intergenic Region A	C	G	T
AAAAAAA	-	28.48%	31.04%	40.48%	-	28.05%	31.29%	40.65%
CCCCCCC	37.93%	-	16.91%	45.16%	37.66%	-	16.80%	45.54%
GGGGGGG	45.22%	16.91%	-	37.87%	45.52%	16.79%	-	37.69%
TTTTTTT	40.41%	31.06%	28.50%	-	40.66%	31.28%	28.06%	-
ACACACA	41.80%	15.85%	18.99%	23.36%	41.94%	15.78%	18.70%	23.58%
AGAGAGA	43.86%	16.79%	21.76%	17.59%	44.29%	16.54%	21.43%	17.74%
ATATATA	39.21%	14.91%	15.44%	30.43%	39.04%	14.95%	15.77%	30.24%
CACACAC	34.04%	19.40%	20.78%	25.78%	34.30%	19.28%	20.60%	25.82%
CGCGCGC	24.41%	29.08%	22.76%	23.75%	23.88%	28.96%	23.24%	23.91%
CTCTCTC	17.49%	25.84%	18.82%	37.86%	17.47%	25.40%	18.70%	38.43%
GAGAGAG	37.89%	18.82%	25.87%	17.43%	38.50%	18.72%	25.34%	17.44%
GCGCGCG	24.29%	22.68%	29.08%	23.96%	23.91%	23.91%	22.99%	29.23%
GTGTGTG	25.71%	20.84%	19.43%	34.02%	25.83%	20.61%	19.26%	34.29%
TATATAT	30.42%	15.45%	14.92%	39.21%	30.24%	15.75%	14.98%	39.02%
TCTCTCT	17.52%	21.77%	16.77%	43.94%	17.75%	21.48%	16.57%	44.19%
TGTGTGT	23.27%	19.02%	15.86%	41.86%	23.59%	18.75%	15.74%	41.91%
mean	32.23%	21.13%	21.13%	32.18%	32.31%	21.02%	21.05%	32.29%
AAAAAAAAAAA	-	29.72%	37.36%	32.91%	-	29.40%	37.73%	32.86%
CCCCCCCCCCC	40.11%	-	19.32%	40.56%	38.89%	-	19.13%	41.98%
GGGGGGGGGGG	41.16%	19.42%	-	39.42%	41.57%	19.31%	-	39.12%
TTTTTTTTTTT	32.78%	37.44%	29.78%	-	33.01%	37.70%	29.29%	-
ACACACACACA	29.68%	14.15%	24.26%	31.91%	29.95%	13.88%	23.69%	32.48%
AGAGAGAGAGA	42.99%	17.03%	27.46%	12.52%	43.73%	16.82%	26.98%	12.48%
ATATATATATA	28.64%	20.88%	21.22%	29.26%	28.40%	20.84%	21.49%	29.27%
CACACACACAC	29.89%	11.88%	23.41%	34.83%	30.98%	11.51%	22.91%	34.60%
CGCGCGCGCGC	33.67%	17.94%	16.99%	31.40%	32.56%	20.27%	18.52%	28.65%
CTCTCTCTCTC	12.31%	28.82%	17.55%	41.32%	12.26%	28.13%	17.22%	42.39%
GAGAGAGAGAG	41.93%	17.19%	28.78%	12.10%	42.78%	17.18%	28.09%	11.94%
GCGCGCGCGCG	31.17%	16.91%	18.14%	33.78%	29.61%	16.89%	20.61%	32.89%
GTGTGTGTGTG	33.92%	23.03%	12.74%	30.31%	34.60%	23.14%	11.48%	30.78%
TATATATATAT	29.22%	21.34%	20.79%	28.65%	29.21%	21.55%	20.83%	28.41%
TCTCTCTCTCT	12.84%	27.56%	17.27%	42.33%	12.74%	26.91%	16.99%	43.37%
TGTGTGTGTGT	31.39%	23.95%	14.51%	30.15%	32.57%	23.96%	13.66%	29.81%
mean	31.45%	21.82%	21.97%	31.43%	31.52%	21.83%	21.91%	32.40%

## Supplementary Materials

The following are available online at http://www.mdpi.com/2073-4425/9/10/482/s1, Figure S1: Heatmap of Correlations between individual intron and intergenic regions (k = 11)., Figure S2: Markov Simulation (order: zero, k=10), Figure S3: Markov Simulation (order: zero, k=7), Figure S4: Markov Simulation (order: 1, k=7), Figure S5: Markov Simulation (order: 2, k=7), Table S1: Accession numbers of sequences, Table S2: List of top 100 k-mer words with highest contribution to correlation values for k = 7, Table S3: Table S3. List of 100 k-mer words with highest contribution to correlation values for k = 11.

## Aknowledgements

We acknowledge financial support by Deutsche Forschungsgemeinschaft within the funding programme Open Access Publishing, by the Baden-Württemberg Ministry of Science, Research and the Arts and by Ruprecht-Karls-Universität Heidelberg.
