# Supplementary material for: Conservation of k-mer Composition and Correlation Contribution between Introns and Intergenic Regions of Animalia Genomes
Source: Genes (Basel). 2018 Oct 4;9(10):482. doi: 10.3390/genes9100482 (PMC6211125; doi:10.3390/genes9100482)
Supplement: Supplementary file 1 [file genes-09-00482-s001.pdf]

Supplementary Materials:

Table S1. Accession Numbers of Sequences.

Species	Accession Numbers
Homo sapiens	NC_000001.11, NC_000002.12, NC_000003.12, NC_000004.12, NC_000005.10, NC_000006.12, NC_000007.14, NC_000008.11, NC_000009.12, NC_000010.11, NC_000011.10, NC_000012.12, NC_000013.11, NC_000014.9, NC_000015.10, NC_000016.10, NC_000017.11, NC_000018.10, NC_000019.10, NC_000020.11, NC_000021.9, NC_000022.11, NC_000023.11, NC_000024.10
Pan troglodytes	NC_006468.4, NC_006469.4, NC_006470.4, NC_006490.4, NC_006471.4, NC_006472.4, NC_006473.4, NC_006474.4, NC_006475.4, NC_006476.4, NC_006477.4, NC_006478.4, NC_006479.4, NC_006480.4, NC_006481.4, NC_006482.4, NC_006483.4, NC_006484.4, NC_006485.4, NC_006486.4, NC_006487.4, NC_006488.4, NC_006489.4, NC_006491.4, NC_006492.4
Mus musculus	NC_000067.6, NC_000068.7, NC_000069.6, NC_000070.6, NC_000071.6, NC_000072.6, NC_000073.6, NC_000074.6, NC_000075.6, NC_000076.6, NC_000077.6, NC_000078.6, NC_000079.6, NC_000080.6, NC_000081.6, NC_000082.6, NC_000083.6, NC_000084.6, NC_000085.6, NC_000086.7, NC_000087.7
Rattus norvegicus	NC_005100.4, NC_005101.4, NC_005102.4, NC_005103.4, NC_005104.4, NC_005105.4, NC_005106.4, NC_005107.4, NC_005108.4, NC_005109.4, NC_005110.4, NC_005111.4, NC_005112.4, NC_005113.4, NC_005114.4, NC_005115.4, NC_005116.4, NC_005117.4, NC_005118.4, NC_005119.4, NC_005120.4, NC_024475.1
Oryctolagus cuniculus	NC_013690.1, NC_013669.1, NC_013670.1, NC_013671.1, NC_013672.1, NC_013673.1, NC_013674.1, NC_013675.1, NC_013676.1, NC_013677.1, NC_013678.1, NC_013679.1, NC_013680.1, NC_013681.1, NC_013682.1, NC_013683.1, NC_013684.1, NC_013685.1, NC_013686.1, NC_013687.1, NC_013688.1, NC_013689.1
Canis lupus	NC_006583.3, NC_006584.3, NC_006585.3, NC_006586.3, NC_006587.3, NC_006588.3, NC_006589.3, NC_006590.3, NC_006591.3, NC_006592.3, NC_006593.3, NC_006594.3, NC_006595.3, NC_006596.3, NC_006597.3, NC_006598.3, NC_006599.3, NC_006600.3, NC_006601.3, NC_006602.3, NC_006603.3, NC_006604.3, NC_006605.3, NC_006606.3, NC_006607.3, NC_006608.3, NC_006609.3, NC_006610.3, NC_006611.3, NC_006612.3, NC_006613.3, NC_006614.3, NC_006615.3, NC_006616.3, NC_006617.3, NC_006618.3, NC_006619.3, NC_006620.3, NC_006621.3
Felis catus	NC_018723.2, NC_018724.2, NC_018725.2, NC_018726.2, NC_018727.2, NC_018728.2, NC_018729.2, NC_018730.2, NC_018731.2, NC_018732.2, NC_018733.2, NC_018734.2, NC_018735.2, NC_018736.2, NC_018737.2, NC_018738.2, NC_018739.2, NC_018740.2, NC_018741.2
Equus caballus	NC_009144.2, NC_009145.2, NC_009146.2, NC_009147.2, NC_009148.2, NC_009149.2, NC_009150.2, NC_009151.2, NC_009152.2, NC_009153.2, NC_009154.2, NC_009155.2, NC_009156.2, NC_009157.2, NC_009158.2, NC_009159.2, NC_009160.2, NC_009161.2, NC_009162.2, NC_009163.2, NC_009164.2, NC_009165.2, NC_009166.2, NC_009167.2, NC_009168.2, NC_009169.2, NC_009170.2, NC_009171.2, NC_009172.2, NC_009173.2, NC_009174.2, NC_009175.2
Capra hircus	NC_030808.1, NC_030809.1, NC_030810.1, NC_030811.1, NC_030812.1, NC_030813.1, NC_030814.1, NC_030815.1, NC_030816.1, NC_030817.1, NC_030818.1, NC_030819.1, NC_030820.1, NC_030821.1, NC_030822.1, NC_030823.1, NC_030824.1, NC_030825.1, NC_030826.1, NC_030827.1, NC_030828.1, NC_030829.1, NC_030830.1, NC_030831.1, NC_030832.1, NC_030833.1, NC_030834.1, NC_030835.1, NC_030836.1
Bos taurus	AC_000158.1, AC_000159.1, AC_000160.1, AC_000161.1, AC_000162.1, AC_000163.1, AC_000164.1, AC_000165.1, AC_000166.1, AC_000167.1, AC_000168.1, AC_000169.1, AC_000170.1, AC_000171.1, AC_000172.1, AC_000173.1, AC_000174.1, AC_000175.1, AC_000176.1, AC_000177.1, AC_000178.1, AC_000179.1, AC_000180.1, AC_000181.1, AC_000182.1, AC_000183.1, AC_000184.1, AC_000185.1, AC_000186.1, AC_000187.1
Sus scrofa	NC_010443.5, NC_010444.4, NC_010445.4, NC_010446.5, NC_010447.5, NC_010448.4, NC_010449.5, NC_010450.4, NC_010451.4, NC_010452.4, NC_010453.5, NC_010454.4, NC_010455.5, NC_010456.5, NC_010457.5, NC_010458.4, NC_010459.5, NC_010460.4, NC_010461.5, NC_010462.3
Monodelphis domestica	NC_008801.1, NC_008802.1, NC_008803.1, NC_008804.1, NC_008805.1, NC_008806.1, NC_008807.1, NC_008808.1, NC_008809.1
Ornithorhynchus anaticus	NC_009094.1, NC_009095.1, NC_009096.1, NC_009097.1, NC_009098.1, NC_009099.1, NC_009100.1, NC_009103.1, NC_009104.1, NC_009105.1, NC_009107.1, NC_009108.1, NC_009110.1, NC_009111.1, NC_009112.1, NC_009114.1, NC_009115.1, NC_009116.1, NC_009118.1
Gallus gallus	NC_006088.4, NC_006089.4, NC_006090.4, NC_006091.4, NC_006092.4, NC_006093.4, NC_006094.4, NC_006095.4, NC_006096.4, NC_006097.4, NC_006098.4, NC_006099.4, NC_006100.4, NC_006101.4, NC_006102.4, NC_006103.4, NC_006104.4, NC_006105.4,

	NC_006106.4, NC_006107.4, NC_006108.4, NC_006109.4, NC_006110.4, NC_006111.4, NC_006112.3, NC_006113.4, NC_006114.4, NC_006115.4, NC_028739.1, NC_028740.1, NC_006119.3, NC_008465.3, NC_006126.4, NC_006127.4
Taeniopygia guttata	NC_011462.1, NC_011463.1, NC_011464.1, NC_011465.1, NC_011466.1, NC_011467.1, NC_011468.1, NC_011469.1, NC_011470.1, NC_011471.1, NC_011472.1, NC_011473.1, NC_011474.1, NC_011475.1, NC_011476.1, NC_011477.1, NC_011478.1, NC_011479.1, NC_011480.1, NC_011481.1, NC_011482.1, NC_011483.1, NC_011484.1, NC_011485.1, NC_011486.1, NC_011487.1, NC_011488.1, NC_011489.1, NC_011490.1, NC_011491.1, NC_011492.1, NC_011496.1, NC_011494.1, NC_011495.1, NC_011493.1
Ficedula albicollis	NC_021671.1, NC_021672.1, NC_021673.1, NC_021674.1, NC_021675.1, NC_021676.1, NC_021677.1, NC_021678.1, NC_021679.1, NC_021680.1, NC_021681.1, NC_021682.1, NC_021683.1, NC_021684.1, NC_021685.1, NC_021686.1, NC_021687.1, NC_021688.1, NC_021689.1, NC_021690.1, NC_021691.1, NC_021692.1, NC_021693.1, NC_021694.1, NC_021695.1, NC_021696.1, NC_021697.1, NC_021698.1, NC_021699.1, NC_021700.1
Chrysemys picta	NC_024218.1, NC_024219.1, NC_024220.1, NC_024221.1, NC_024222.1, NC_024223.1, NC_024224.1, NC_024225.1, NC_024226.1, NC_024227.1, NC_024228.1, NC_024229.1, NC_024230.1, NC_024231.1, NC_024232.1, NC_024233.1, NC_024234.1, NC_024235.1
Anolis carolinensis	NC_014776.1, NC_014777.1, NC_014778.1, NC_014779.1, NC_014780.1, NC_014781.1, NC_014782.1, NC_014783.1, NC_014784.1, NC_014785.1, NC_014786.1, NC_014787.1, NC_014788.1
Xenopus tropicalis	NC_030677.1, NC_030678.1, NC_030679.1, NC_030680.1, NC_030681.1, NC_030682.1, NC_030683.1, NC_030684.1, NC_030685.1, NC_030686.1
Takifugu rubripes	NC_018890.1, NC_018891.1, NC_018892.1, NC_018893.1, NC_018894.1, NC_018895.1, NC_018896.1, NC_018897.1, NC_018898.1, NC_018899.1, NC_018900.1, NC_018901.1, NC_018902.1, NC_018903.1, NC_018904.1, NC_018905.1, NC_018906.1, NC_018907.1, NC_018908.1, NC_018909.1, NC_018910.1, NC_018911.1
Cynoglossus semilaevis	NC_024307.1, NC_024308.1, NC_024309.1, NC_024310.1, NC_024311.1, NC_024312.1, NC_024313.1, NC_024314.1, NC_024315.1, NC_024316.1, NC_024317.1, NC_024318.1, NC_024319.1, NC_024320.1, NC_024321.1, NC_024322.1, NC_024323.1, NC_024324.1, NC_024325.1, NC_024326.1, NC_024327.1, NC_024328.1
Oreochromis niloticus	NC_031965.1, NC_031966.1, NC_031967.1, NC_031968.1, NC_031969.1, NC_031970.1, NC_031971.1, NC_031972.1, NC_031973.1, NC_031974.1, NC_031975.1, NC_031976.1, NC_031977.1, NC_031978.1, NC_031979.1, NC_031980.1, NC_031987.1, NC_031981.1, NC_031982.1, NC_031983.1, NC_031984.1, NC_031985.1, NC_031986.1
Poecilia reticulata	NC_024331.1, NC_024332.1, NC_024333.1, NC_024334.1, NC_024335.1, NC_024336.1, NC_024337.1, NC_024338.1, NC_024339.1, NC_024340.1, NC_024341.1, NC_024342.1, NC_024343.1, NC_024344.1, NC_024345.1, NC_024346.1, NC_024347.1, NC_024348.1, NC_024349.1, NC_024350.1, NC_024351.1, NC_024352.1, NC_024353.1
Danio rerio	NC_007112.6, NC_007113.6, NC_007114.6, NC_007115.6, NC_007116.6, NC_007117.6, NC_007118.6, NC_007119.6, NC_007120.6, NC_007121.6, NC_007122.6, NC_007123.6, NC_007124.6, NC_007125.6, NC_007126.6, NC_007127.6, NC_007128.6, NC_007129.6, NC_007130.6, NC_007131.6, NC_007132.6, NC_007133.6, NC_007134.6, NC_007135.6, NC_007136.6
Salmo salar	NC_027300.1, NC_027301.1, NC_027302.1, NC_027303.1, NC_027304.1, NC_027305.1, NC_027306.1, NC_027307.1, NC_027308.1, NC_027309.1, NC_027310.1, NC_027311.1, NC_027312.1, NC_027313.1, NC_027314.1, NC_027315.1, NC_027316.1, NC_027317.1, NC_027318.1, NC_027319.1, NC_027320.1, NC_027321.1, NC_027322.1, NC_027323.1, NC_027324.1, NC_027325.1, NC_027326.1, NC_027327.1, NC_027328.1
Lepisosteus oculatus	NC_023179.1, NC_023180.1, NC_023181.1, NC_023182.1, NC_023183.1, NC_023184.1, NC_023185.1, NC_023186.1, NC_023187.1, NC_023188.1, NC_023189.1, NC_023190.1, NC_023191.1, NC_023192.1, NC_023193.1, NC_023194.1, NC_023195.1, NC_023196.1, NC_023197.1, NC_023198.1, NC_023199.1, NC_023200.1, NC_023201.1, NC_023202.1, NC_023203.1, NC_023204.1, NC_023205.1, NC_023206.1, NC_023207.1
Ciona intestinalis	NC_020166.2, NC_020167.2, NC_020168.2, NC_020169.2, NC_020170.2, NC_020171.2, NC_020172.2, NC_020173.2, NC_020174.2, NC_020175.2, NC_020176.2, NC_020177.2, NC_020178.2, NC_020179.2
Schistosoma mansoni	NC_031496.1, NC_031497.1, NC_031498.1, NC_031499.1, NC_031500.1, NC_031501.1
Strongyloides ratti	LN609528.1, LN609529.1, LN609530.1
Caenorhabditis brigsae	FR847112.2, FR847113.2, FR847114.2, FR847118.2, FR847121.2, FR847123.2
Caenorhabditis elegans	NC_003279.8, NC_003280.10, NC_003281.10, NC_003282.8, NC_003283.11, NC_003284.9
Apis mellifera	NC_007070.3, NC_007071.3, NC_007072.3, NC_007073.3, NC_007074.3, NC_007075.3, NC_007076.3, NC_007077.3, NC_007078.3, NC_007079.3, NC_007080.3, NC_007081.3, NC_007082.3, NC_007083.3, NC_007084.3, NC_007085.3

Bombus terrestris	NC_015762.1, NC_015763.1, NC_015764.1, NC_015765.1, NC_015766.1, NC_015767.1, NC_015768.1, NC_015769.1, NC_015770.1, NC_015771.1, NC_015772.1, NC_015773.1, NC_015774.1, NC_015775.1, NC_015776.1, NC_015777.1, NC_015778.1, NC_015779.1
Nasonia vitripennis	NC_015867.2, NC_015868.2, NC_015869.2, NC_015870.2, NC_015871.2
Anopheles gambiae	NT_078265.2, NT_078267.5, NC_004818.2, NT_078266.2, NT_078268.4
Drosphila pseudoobscura	NC_009005.2, NC_009006.2
Drosophila melanogaster	NC_004354.4, NT_033779.5, NT_033778.4, NT_037436.4, NT_033777.3, NC_004353.4, NC_024512.1
Drosohpila simulans	NT_479533.1, NT_479534.1, NT_479535.1, NT_479536.1, NC_029796.1, NC_029795.1

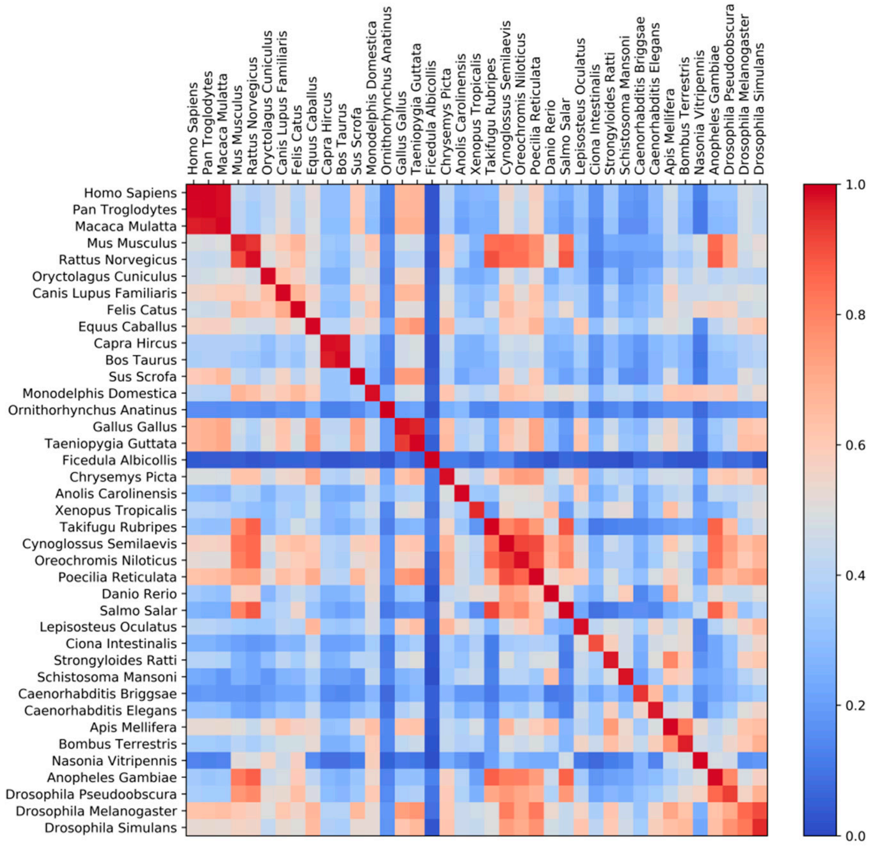

Figure S1 Heatmap of Correlations between individual intron and intergenic regions (k = 11). Each row represents the pairwise correlation values (Pearson correlation) of introns for the listed organism, while every column is associated with the intergenic region of an organism. Color scale limited in heatmap to values above zero for better readability.

Table S2. List of top 100 *k*-mer words with highest contribution to correlation values for k = 7. "Organism Content", shown for homogenous pairings of sequence regions, is defined by the fraction of analyzed organisms for which the difference between content of the corresponding *k*-mer word and the mean *k*-mer word content (4^{-k}) is larger than 1 σ with respect to the *k*-mer word content distribution of the respective organism.

Correlated Regions	*k*-mer word	Contribution	Organism Content
**Exons - Exons**	TTTTTTT	0.0204	100%
	AAAAAAA	0.0196	100%
	GCTGCTG	0.0045	92.3%
	CAGCAGC	0.0044	89.7%
	CTGCTGC	0.0032	82.1%
	GCAGCAG	0.0032	84.6%
	TTTTCTT	0.0029	100%

TTTCTTT	0.0028	100%
TTTATTT	0.0028	97.4%
TGCTGCT	0.0027	92.3%
AAGAAAA	0.0027	100%
AGCAGCA	0.0026	97.4%
AAAGAAA	0.0026	100%
ATTTTTT	0.0024	100%
AAATAAA	0.0023	97.4%
TTTAAAA	0.0023	92.3%
TTTTAAA	0.0023	92.3%
TTTTTCT	0.0021	100%
GGAGGAG	0.0021	92.3%
AAAAAAT	0.0021	100%
CTCCTCC	0.0021	92.3%
TTCTTTT	0.0020	100%
TATTTTT	0.0020	100%
AGAAAAA	0.0020	100%
TTATTTT	0.0020	100%
TTTTATT	0.0020	97.4%
TTTTTAA	0.0019	92.3%
TTAAAAA	0.0019	92.3%
AAAAGAA	0.0019	100%
AAAAATA	0.0019	100%
TTCTTCT	0.0019	100%
AGAAGAA	0.0018	100%
TTTGTTT	0.0018	100%
AAAATAA	0.0018	100%
AAACAAA	0.0017	100%
GAGGAGG	0.0017	87.2%
AATAAAA	0.0017	97.4%
CCTCCTC	0.0017	89.7%
TCTTCTT	0.0016	100%
TTTTTTA	0.0016	92.3%
TTGTTTT	0.0016	100%
CTTCTTC	0.0016	100%
AAGAAGA	0.0016	100%
GAAGAAG	0.0016	100%
AAAACAA	0.0016	100%
AGGAGGA	0.0015	89.7%
TCCTCCT	0.0015	89.7%
CTGCAGC	0.0015	84.6%
GCTGCAG	0.0015	84.6%
TAAAAAA	0.0015	94.9%
TTTTTTC	0.0014	97.4%
ACACACA	0.0014	87.2%
TCTTTTT	0.0014	97.4%
TTTTGTT	0.0014	100%
TGTTTTT	0.0014	100%
TGTGTGT	0.0014	87.2%
AAAAACA	0.0013	100%
AACAAAA	0.0013	100%
GAAAAAA	0.0013	97.4%
TTTCTTC	0.0013	97.4%
ATATTTT	0.0013	100%
CCAGCAG	0.0013	82.1%
AAAAAGA	0.0013	100%
CTGCTGG	0.0013	84.6%
GAAGAAA	0.0013	94.9%
AAAATAT	0.0012	100%
TTCTTCA	0.0012	100%
TTTTTAT	0.0012	100%
TGAAGAA	0.0012	100%

	GCAGCTG	0.0012	84.6%
	CTTTTTT	0.0012	97.4%
	CAGCTGC	0.0012	84.6%
	CACACAC	0.0011	87.2%
	AATATTT	0.0011	100%
	AAAAAAG	0.0011	97.4%
	ATAAAAA	0.0011	100%
	GTGTGTG	0.0011	87.2%
	AAATATT	0.0011	100%
	TTTTTGT	0.0011	100%
	ACAAAAA	0.0010	100%
	TATATAT	0.0010	87.2%
	ATTTTTA	0.0010	94.9%
	ATATATA	0.0010	87.2%
	TAAAAAT	0.0010	94.9%
	ATTTATT	0.0009	97.4%
	GGAGCTG	0.0009	87.2%
	CAGCTCC	0.0009	87.2%
	AATTTTT	0.0009	89.7%
	TTTTTCA	0.0009	94.9%
	CATTTTT	0.0009	97.4%
	AGCTGCT	0.0009	87.2%
	CTCCAGC	0.0009	84.6%
	GCTGGAG	0.0009	84.6%
	AGCAGCT	0.0009	84.6%
	AAAAATT	0.0009	89.7%
	AAAAATG	0.0009	97.4%
	TGAAAAA	0.0009	97.4%
	ATTTTCT	0.0008	94.9%
	CTTCTCC	0.0008	92.3%
	TCTTCCT	0.0008	92.3%
**CDS - CDS**	GCTGCTG	0.0077	94.9%
	CAGCAGC	0.0077	94.9%
	CTGCTGC	0.0054	92.3%
	GCAGCAG	0.0053	89.7%
	TGCTGCT	0.0042	94.9%
	AGCAGCA	0.0041	94.9%
	CTCCTCC	0.0035	89.7%
	GGAGGAG	0.0035	89.7%
	CTTCTTC	0.0031	100%
	GAAGAAG	0.003	100%
	CCTCCTC	0.0028	87.2%
	GAGGAGG	0.0028	87.2%
	CTGCAGC	0.0027	82.1%
	GCTGCAG	0.0027	82.1%
	TTCTTCT	0.0027	100%
	AGAAGAA	0.0027	100%
	CCAGCAG	0.0026	82.1%
	CTGCTGG	0.0026	82.1%
	TCTTCTT	0.0025	100%
	TCCTCCT	0.0025	89.7%
	AAGAAGA	0.0025	100%
	AGGAGGA	0.0025	92.3%
	GCAGCTG	0.0022	82.1%
	CAGCTGC	0.0022	82.1%
	TTCTTCA	0.0020	100%
	TGAAGAA	0.0020	100%
	CAGCTCC	0.0019	87.2%
	GGAGCTG	0.0019	87.2%
	CTCCAGC	0.0018	87.2%
	GCTGGAG	0.0018	87.2%

TGGAGGA	0.0017	94.9%
TCCTCCA	0.0016	94.9%
CTTCTCC	0.0016	92.3%
AGCTGCT	0.0016	84.6%
GGAGAAG	0.0016	92.3%
AGCAGCT	0.0016	87.2%
TTTCTTC	0.0015	92.3%
CCTGCTG	0.0015	82.1%
CAGCAGG	0.0015	82.1%
CTGCAGG	0.0015	84.6%
CAGCTGG	0.0015	84.6%
CCAGCTG	0.0015	84.6%
CCTGCAG	0.0015	79.5%
GAAGAAA	0.0015	92.3%
TTCTCCA	0.0014	100%
TGGAGAA	0.0014	100%
CTGGAGG	0.0014	79.5%
CCTCCAG	0.0014	79.5%
TTTTCTT	0.0014	87.2%
CTGCTCC	0.0013	87.2%
GGAGCAG	0.0013	87.2%
CTTCCTC	0.0013	94.9%
GAGGAAG	0.0013	94.9%
AAGAAAA	0.0013	87.2%
AGGAAGA	0.0012	89.7%
TCTCCAG	0.0012	84.6%
TCTTCCT	0.0012	89.7%
TCCAGCA	0.0012	89.7%
CTGGAGA	0.0012	84.6%
CATCTTC	0.0012	100%
TGCTGGA	0.0012	89.7%
GAAGATG	0.0012	100%
TCTTCAT	0.0012	97.4%
CCTGGAG	0.0012	76.9%
TCCAGCT	0.0012	87.2%
AGCTGGA	0.0012	87.2%
ATGAAGA	0.0012	97.4%
CATCATC	0.0012	100%
CTCCAGG	0.0012	76.9%
GCTGCTC	0.0011	87.2%
GATGATG	0.0011	100%
GAGCAGC	0.0011	87.2%
CCAGCTC	0.0011	84.6%
TTTCTTT	0.0011	87.2%
GAGCTGG	0.0011	84.6%
TCTTCAG	0.0011	100%
CTTCATC	0.0011	100%
GATGAAG	0.0010	100%
CTGAAGA	0.0010	97.4%
TCTTCTG	0.0010	97.4%
AAAGAAA	0.0010	87.2%
CAGAAGA	0.0010	97.4%
GAAGCTG	0.0010	92.3%
CTGGAGC	0.0010	87.2%
TGCAGCA	0.0010	89.7%
CAGCTTC	0.0010	92.3%
TGCTGCA	0.0010	89.7%
TCTGCTG	0.0010	84.6%
GCTCCAG	0.0010	87.2%
CAGCAGA	0.0010	84.6%
CTCCTGG	0.0010	79.5%
CCAGGAG	0.0010	79.5%

	CTCCTTC	0.0009	89.7%
	GAAGAGG	0.0009	92.3%
	CCTCTTC	0.0009	92.3%
	TTCTCCT	0.0009	92.3%
	TCTGCAG	0.0009	82.1%
	GAGAAGA	0.0009	92.3%
	TCTTCTC	0.0009	92.3%
	GAAGGAG	0.0009	89.7%
**Introns -Introns**	TTTTTTT	0.0553	100%
	AAAAAAA	0.0552	100%
	TGTGTGT	0.0070	89.7%
	ACACACA	0.0070	87.2%
	TTTATTT	0.0065	100%
	AAATAAA	0.0064	100%
	ATATATA	0.0063	100%
	TATATAT	0.0063	100%
	ATTTTTT	0.0056	100%
	GTGTGTG	0.0056	82.1%
	AAAAAAT	0.0055	100%
	CACACAC	0.0055	82.1%
	TTTAAAA	0.0052	100%
	TTTTAAA	0.0052	100%
	TATTTTT	0.0048	100%
	AAAAATA	0.0048	100%
	TTTTTAA	0.0047	100%
	TTAAAAA	0.0047	100%
	TTATTTT	0.0047	100%
	AAAATAA	0.0047	100%
	TTTTTTA	0.0041	100%
	TAAAAAA	0.0041	100%
	TTTTATT	0.0040	100%
	AATAAAA	0.0040	100%
	AGAGAGA	0.0037	92.3%
	TCTCTCT	0.0037	89.7%
	TTTCTTT	0.0033	100%
	AAAGAAA	0.0032	100%
	TTTGTTT	0.0029	100%
	AAACAAA	0.0029	100%
	GAGAGAG	0.0029	76.9%
	CTCTCTC	0.0029	76.9%
	ATATTTT	0.0028	100%
	AAAATAT	0.0028	100%
	TTTTTAT	0.0028	100%
	ATAAAAA	0.0027	100%
	TTTTCTT	0.0027	100%
	AAGAAAA	0.0027	100%
	ATTTTTA	0.0027	100%
	TAAAAAT	0.0027	100%
	AATTTTT	0.0026	94.9%
	AAAAATT	0.0026	94.9%
	TTTTTTC	0.0026	100%
	ATTTATT	0.0026	100%
	AATAAAT	0.0026	100%
	GAAAAAA	0.0026	100%
	TTTTTCT	0.0026	100%
	AGAAAAA	0.0025	100%
	AATATTT	0.0024	100%
	AAATATT	0.0024	100%
	TTGTTTT	0.0024	100%
	AAAACAA	0.0024	100%
	TGTTTTT	0.0023	100%

	AAAAACA	0.0023	100%
	CTTTTTT	0.0022	100%
	AAAAAAG	0.0022	100%
	TTCTTTT	0.0021	100%
	AAAAGAA	0.0021	100%
	TTTTAAT	0.0021	100%
	ATTAAAA	0.0021	100%
	TTTTGTT	0.0020	100%
	AACAAAA	0.0020	100%
	TTAAAAT	0.0020	100%
	ATTTTAA	0.0020	100%
	TATTTAT	0.0020	100%
	ATAAATA	0.0020	100%
	AAAATTT	0.0019	94.9%
	AAATTTT	0.0019	94.9%
	TTTAAAT	0.0018	100%
	ATTTAAA	0.0018	100%
	TATTTTA	0.0018	100%
	TAAAATA	0.0018	100%
	TTTTTTG	0.0018	100%
	TCTTTTT	0.0018	100%
	ATTTTAT	0.0018	100%
	CAAAAAA	0.0018	100%
	ATAAAAT	0.0018	100%
	AAAAAGA	0.0018	100%
	TAAATAA	0.0018	100%
	TTATTTA	0.0018	100%
	TAATTTT	0.0018	100%
	AAAATTA	0.0017	100%
	TTTTTGT	0.0017	100%
	ACAAAAA	0.0017	100%
	AAATAAT	0.0017	100%
	ATTATTT	0.0017	100%
	AATTAAA	0.0016	100%
	TTTAATT	0.0016	100%
	CATTTTT	0.0016	100%
	AAAAATG	0.0016	100%
	AATAATA	0.0015	100%
	TATTATT	0.0015	100%
	TTAATTT	0.0015	100%
	AAATTAA	0.0015	100%
	AAATATA	0.0015	100%
	TATATTT	0.0015	100%
	ATAATAA	0.0013	100%
	TTATTAT	0.0013	100%
	AAAATGT	0.0013	100%
	ACATTTT	0.0013	100%
**Intergenic – Intergenic**	TTTTTTT	0.0467	100%
	AAAAAAA	0.0466	100%
	ATATATA	0.0073	100%
	TATATAT	0.0072	100%
	AAATAAA	0.0069	100%
	TTTATTT	0.0069	100%
	TGTGTGT	0.0059	89.7%
	ACACACA	0.0057	89.7%
	ATTTTTT	0.0055	100%
	AAAAAAT	0.0055	100%
	TATTTTT	0.005	100%
	AAAAATA	0.0049	100%
	TTATTTT	0.0048	100%
	TTTAAAA	0.0047	100%

AAAATAA	0.0047	100%
TTTTAAA	0.0046	100%
GTGTGTG	0.0046	82.1%
CACACAC	0.0045	82.1%
TTAAAAA	0.0044	100%
AATAAAA	0.0043	100%
TTTTTAA	0.0043	100%
TTTTATT	0.0042	100%
TAAAAAA	0.0038	100%
TTTTTTA	0.0037	100%
AGAGAGA	0.0037	87.2%
TCTCTCT	0.0037	84.6%
AAAGAAA	0.0036	100%
TTTCTTT	0.0035	100%
AAAATAT	0.0031	100%
ATATTTT	0.0031	100%
AATAAAT	0.0030	100%
TTTTCTT	0.0029	100%
TTTTTAT	0.0029	100%
ATAAAAA	0.0029	100%
AAGAAAA	0.0029	100%
AAACAAA	0.0029	100%
GAGAGAG	0.0028	79.5%
CTCTCTC	0.0028	79.5%
TTTGTTT	0.0028	100%
ATTTATT	0.0028	100%
AAAAATT	0.0028	97.4%
AATTTTT	0.0028	97.4%
AAATATT	0.0027	100%
TAAAAAT	0.0027	100%
ATTTTTA	0.0027	100%
TTTTTCT	0.0026	100%
AGAAAAA	0.0026	100%
AATATTT	0.0026	100%
GAAAAAA	0.0025	100%
TTTTTTC	0.0025	100%
TTGTTTT	0.0023	100%
AAAACAA	0.0023	100%
AAAAGAA	0.0023	97.4%
TTCTTTT	0.0022	97.4%
AAATTTT	0.0022	92.3%
TTTTAAT	0.0022	100%
ATAAATA	0.0022	100%
TGTTTTT	0.0021	100%
TATTTAT	0.0021	100%
AAAATTT	0.0021	94.9%
ATTAAAA	0.0021	100%
AAAAACA	0.0021	100%
ATTTTAA	0.0021	100%
TTTTGTT	0.0021	100%
CTTTTTT	0.0020	100%
AAAAAAG	0.0020	100%
AACAAAA	0.0020	100%
TAAAATA	0.0019	100%
ATTTTAT	0.0019	100%
ATAAAAT	0.0019	100%
TTAAAAT	0.0019	100%
TTTTTTG	0.0018	100%
ATTATTT	0.0018	100%
TATTTTA	0.0018	100%
AATTAAA	0.0018	100%
TTATTTA	0.0018	100%

	TAAATAA	0.0018	100%
	AAAATTA	0.0018	100%
	TAATTTT	0.0018	100%
	ATTTAAA	0.0018	100%
	CAAAAAA	0.0018	100%
	TCTTTTT	0.0018	100%
	AAAAAGA	0.0018	100%
	AAATAAT	0.0017	100%
	TTTAAAT	0.0017	100%
	TTTTTGT	0.0017	100%
	TTTAATT	0.0017	100%
	AATAATA	0.0017	100%
	TATTATT	0.0017	100%
	ACAAAAA	0.0017	100%
	AAATATA	0.0017	100%
	TATATTT	0.0017	100%
	AAATTAA	0.0016	100%
	CCCCCCC	0.0016	38.5%
	CATTTTT	0.0016	100%
	AAAAATG	0.0016	100%
	ATAATAA	0.0015	100%
	TTATTAT	0.0015	100%
	TTAATTT	0.0015	100%
	AATTTTA	0.0013	92.3%
**Introns - Intergenic**	TTTTTTT	0.0510	
	AAAAAAA	0.0510	
	ATATATA	0.0068	
	TATATAT	0.0068	
	AAATAAA	0.0066	
	TTTATTT	0.0066	
	TGTGTGT	0.0064	
	ACACACA	0.0063	
	ATTTTTT	0.0056	
	AAAAAAT	0.0056	
	GTGTGTG	0.0051	
	CACACAC	0.0050	
	TTTAAAA	0.0049	
	TTTTAAA	0.0049	
	TATTTTT	0.0049	
	AAAAATA	0.0049	
	TTATTTT	0.0047	
	AAAATAA	0.0047	
	TTAAAAA	0.0046	
	TTTTTAA	0.0045	
	AATAAAA	0.0041	
	TTTTATT	0.0041	
	TAAAAAA	0.0040	
	TTTTTTA	0.0040	
	AGAGAGA	0.0038	
	TCTCTCT	0.0038	
	AAAGAAA	0.0034	
	TTTCTTT	0.0034	
	GAGAGAG	0.0030	
	AAAATAT	0.0030	
	CTCTCTC	0.0030	
	ATATTTT	0.0030	
	AAACAAA	0.0029	
	TTTGTTT	0.0029	
	TTTTTAT	0.0029	
	ATAAAAA	0.0028	
	TTTTCTT	0.0028	

AATTTTT	0.0028
AAAAATT	0.0028
AAGAAAA	0.0028
AATAAAT	0.0028
ATTTTTA	0.0027
TAAAAAT	0.0027
ATTTATT	0.0027
TTTTTCT	0.0026
AGAAAAA	0.0026
AAATATT	0.0026
TTTTTTC	0.0026
GAAAAAA	0.0026
AATATTT	0.0025
TTGTTTT	0.0023
AAAACAA	0.0023
TGTTTTT	0.0022
AAAAGAA	0.0022
TTCTTTT	0.0022
AAAAACA	0.0022
TTTTAAT	0.0021
AAATTTT	0.0021
CTTTTTT	0.0021
AAAAAAG	0.0021
ATTAAAA	0.0021
AAAATTT	0.0021
ATAAATA	0.0020
TTTTGTT	0.0020
TATTTAT	0.0020
ATTTTAA	0.0020
AACAAAA	0.0020
TTAAAAT	0.0019
TAAAATA	0.0019
TTTTTTG	0.0018
ATTTTAT	0.0018
ATAAAAT	0.0018
TATTTTA	0.0018
ATTTAAA	0.0018
CAAAAAA	0.0018
TTTAAAT	0.0018
TAATTTT	0.0018
AAAATTA	0.0018
TTATTTA	0.0018
TAAATAA	0.0018
TCTTTTT	0.0018
AAAAAGA	0.0018
ATTATTT	0.0018
TTTTTGT	0.0017
AATTAAA	0.0017
AAATAAT	0.0017
ACAAAAA	0.0017
CCCCCCC	0.0017
TTTAATT	0.0017
AATAATA	0.0016
TATTATT	0.0016
AAATATA	0.0016
CATTTTT	0.0016
AAAAATG	0.0016
TATATTT	0.0016
AAATTAA	0.0016
TTAATTT	0.0015
GGGGGGG	0.0015
ATAATAA	0.0014

**Exons - Introns**	TTTTTTT	0.0414
	AAAAAAA	0.0406
	TTTATTT	0.0052
	AAATAAA	0.0047
	ATTTTTT	0.0044
	TTTAAAA	0.0042
	TTTTAAA	0.0042
	AAAAAAT	0.0041
	ACACACA	0.004
	TGTGTGT	0.0039
	TATTTTT	0.0038
	TTATTTT	0.0037
	TTTTTAA	0.0036
	TTTCTTT	0.0036
	TTAAAAA	0.0036
	AAAAATA	0.0036
	AAAATAA	0.0035
	AAAGAAA	0.0035
	TTTTATT	0.0034
	TTTTCTT	0.0033
	AAGAAAA	0.0032
	TATATAT	0.0032
	CACACAC	0.0032
	AATAAAA	0.0032
	ATATATA	0.0032
	TTTTTTA	0.0031
	GTGTGTG	0.0031
	TAAAAAA	0.003
	TTTTTCT	0.0028
	TTTGTTT	0.0028
	AAACAAA	0.0027
	AGAAAAA	0.0027
	TTCTTTT	0.0025
	TTGTTTT	0.0024
	AAAAGAA	0.0024
	AAAACAA	0.0023
	ATATTTT	0.0023
	TTTTTTC	0.0023
	AAAATAT	0.0022
	TTTTTAT	0.0022
	GAAAAAA	0.0022
	AGAGAGA	0.0022
	TCTCTCT	0.0022
	TGTTTTT	0.0021
	AAAAACA	0.0021
	ATAAAAA	0.0021
	TTTTGTT	0.002
	AATATTT	0.002
	AACAAAA	0.002
	AAATATT	0.002
	ATTTTTA	0.0019
	TAAAAAT	0.0019
	CTTTTTT	0.0019
	ATTTATT	0.0019
	AATTTTT	0.0019
	TCTTTTT	0.0019
	AAAAAAG	0.0019
	AAAAATT	0.0018
	AAAAAGA	0.0018
	AATAAAT	0.0018
	CTCTCTC	0.0017

	GAGAGAG	0.0017
	TTTTTGT	0.0016
	ACAAAAA	0.0016
	TTTTAAT	0.0016
	ATTAAAA	0.0015
	ATTTTAA	0.0015
	ATTTAAA	0.0015
	TTTTTTG	0.0014
	TTTAAAT	0.0014
	CATTTTT	0.0014
	ATTTTAT	0.0014
	AAATTTT	0.0014
	TTAAAAT	0.0014
	AAAATTT	0.0014
	CAAAAAA	0.0014
	AAAAATG	0.0014
	TATTTAT	0.0013
	TTTTTCA	0.0013
	ATTATTT	0.0013
	TAATTTT	0.0013
	AAATAAT	0.0013
	ATAAAAT	0.0013
	TATTTTA	0.0013
	TGAAAAA	0.0012
	CAGCAGC	0.0012
	GCTGCTG	0.0012
	AAAATTA	0.0012
	TAAAATA	0.0012
	TTTAATT	0.0012
	TTCATTT	0.0012
	ATAAATA	0.0011
	AAAATGT	0.0011
	ACATTTT	0.0011
	TTATTTA	0.0011
	AAATGAA	0.0011
	TATATTT	0.0011
	ATTTTCT	0.0011
	AATTAAA	0.0011
**Exons - CDS**	GCTGCTG	0.0065
	CAGCAGC	0.0065
	CTGCTGC	0.0047
	GCAGCAG	0.0046
	TGCTGCT	0.0037
	AGCAGCA	0.0037
	GGAGGAG	0.003
	CTCCTCC	0.003
	TTCTTCT	0.0025
	AGAAGAA	0.0025
	CTTCTTC	0.0025
	GAAGAAG	0.0025
	CCTCCTC	0.0024
	GAGGAGG	0.0024
	TCTTCTT	0.0023
	AAGAAGA	0.0023
	CTGCAGC	0.0022
	GCTGCAG	0.0022
	TTTTCTT	0.0022
	TCCTCCT	0.0022
	AGGAGGA	0.0022
	AAGAAAA	0.0021
	TTTTTTT	0.0021

CCAGCAG	0.0021
AAAAAAA	0.0021
CTGCTGG	0.0020
TTTCTTT	0.0019
AAAGAAA	0.0018
GCAGCTG	0.0018
CAGCTGC	0.0018
TTCTTCA	0.0018
TGAAGAA	0.0017
TTTCTTC	0.0016
GAAGAAA	0.0016
TTCTTTT	0.0015
CAGCTCC	0.0015
GGAGCTG	0.0015
TTTTTCT	0.0015
AGAAAAA	0.0014
CTCCAGC	0.0014
AAAAGAA	0.0014
GCTGGAG	0.0014
AGCTGCT	0.0013
AGCAGCT	0.0013
TGGAGGA	0.0013
CTTCTCC	0.0013
TCCTCCA	0.0013
GGAGAAG	0.0013
CCTGCTG	0.0012
CAGCAGG	0.0012
CAGCTGG	0.0012
CCAGCTG	0.0012
CTGCAGG	0.0012
CCTGCAG	0.0012
TTCTCCA	0.0012
TGGAGAA	0.0012
TCTTCCT	0.0011
CTTCCTC	0.0011
GAGGAAG	0.0011
AGGAAGA	0.0011
CTGCTCC	0.0011
GGAGCAG	0.0011
CTGGAGG	0.0011
CCTCCAG	0.0010
TCTTCAT	0.0010
ATGAAGA	0.0010
TCTCCAG	0.0010
CTGGAGA	0.0010
TCTTTTT	0.0010
TCCAGCA	0.0010
CATCTTC	0.0009
GCTGCTC	0.0009
TGCTGGA	0.0009
AGCTGGA	0.0009
TCCAGCT	0.0009
GAAGATG	0.0009
GAGCAGC	0.0009
AAAAAGA	0.0009
CATCATC	0.0009
TCTTCTG	0.0009
CCTGGAG	0.0009
CAGAAGA	0.0009
GATGATG	0.0009
CTCCAGG	0.0009
CTTCTTT	0.0009

TCTTCAG	0.0009
ATTTTTT	0.0009
TCTGCTG	0.0009
CAGCAGA	0.0009
CTGAAGA	0.0009
TTCTCCT	0.0009
AGGAGAA	0.0008
TGCAGCA	0.0008
AAAGAAG	0.0008
CCAGCTC	0.0008
GAGCTGG	0.0008
CTGCTGT	0.0008
TGCTGCA	0.0008
GAGAAGA	0.0008

Table S3. List of 100 *k*-mer words with highest contribution to correlation values for k = 11. "Organism Content", shown for homogenous pairings of sequence regions, is defined by the fraction of analyzed organisms for which the difference between content of the corresponding *k*-mer word and the mean *k*-mer word content (4^{-k}) is larger than 1 σ with respect to the *k*-mer word content distribution of the respective organism.

Correlated Regions	*k*-mer word	Contribution	Organism Content
**Exons - Exons**	TTTTTTTTTTT	0.0662	100%
	AAAAAAAAAAA	0.0657	100%
	ACACACACACA	0.0155	97.4%
	CACACACACAC	0.0148	97.4%
	TGTGTGTGTGT	0.0148	92.3%
	GTGTGTGTGTG	0.0142	94.9%
	ATATATATATA	0.0069	97.4%
	TATATATATAT	0.0068	97.4%
	TGCTGCTGCTG	0.0052	100%
	CAGCAGCAGCA	0.0052	100%
	GCTGCTGCTGC	0.0041	100%
	GCAGCAGCAGC	0.0041	100%
	CTGCTGCTGCT	0.0040	100%
	AGCAGCAGCAG	0.0040	100%
	TCTCTCTCTCT	0.0036	97.4%
	AGAGAGAGAGA	0.0036	97.4%
	CTCTCTCTCTC	0.0034	94.9%
	GAGAGAGAGAG	0.0034	94.9%
	GAGGAGGAGGA	0.0024	100%
	TCCTCCTCCTC	0.0024	100%
	GGAGGAGGAGG	0.0019	100%
	CCTCCTCCTCC	0.0019	100%
	AGGAGGAGGAG	0.0017	100%
	CTCCTCCTCCT	0.0017	100%
	TAAAAAAAAAA	0.0016	94.9%
	TTTTTTTTTTA	0.0016	94.9%
	AAAAAAAAAAG	0.0015	97.4%
	CTTTTTTTTTT	0.0015	97.4%
	GGCGGCGGCGG	0.0012	92.3%
	GCGGCGGCGGC	0.0012	92.3%
	CCGCCGCCGCC	0.0012	92.3%
	TTTTTTTTTAA	0.0011	100%
	GCCGCCGCCGC	0.0011	92.3%
	TTAAAAAAAAA	0.0011	100%
	GGGGGGGGGGG	0.0010	92.3%
	GAAAAAAAAAA	0.0010	100%
	TTTTTTTTTTC	0.0009	100%
	ATTTTTTTTTT	0.0009	100%

AAAAAAAAAAT	0.0009	100%
CCCCCCCCCCC	0.0008	89.7%
CGGCGGCGGCG	0.0008	89.7%
CGCCGCCGCCG	0.0007	89.7%
AAAAAAAAAAC	0.0007	100%
TTTTTCTTTTT	0.0007	100%
AAAAAGAAAAA	0.0007	100%
GTTTTTTTTTT	0.0007	100%
TTTTTTTTTTG	0.0007	100%
CAAAAAAAAAA	0.0006	100%
AAAAAAGAAAA	0.0006	100%
TTTTCTTTTTT	0.0006	100%
TCTTTTTTTTT	0.0006	100%
AAAAAAAAAGA	0.0006	100%
TTGTTGTTGTT	0.0006	100%
AGAAAAAAAAA	0.0006	100%
AAGAAGAAGAA	0.0006	100%
AACAACAACAA	0.0006	100%
TGTTGTTGTTG	0.0006	100%
TTTTTTTTTCT	0.0006	100%
TTTTTTCTTTT	0.0005	100%
TTTCTTTTTTT	0.0005	100%
AAAAGAAAAAA	0.0005	100%
AAGAAAAAAAA	0.0005	100%
TTTTTTTCTTT	0.0005	100%
AAAGAAAAAAA	0.0005	100%
TTCTTCTTCTT	0.0005	100%
AAAAAAAGAAA	0.0005	100%
TTTTTTTTCTT	0.0005	100%
CAACAACAACA	0.0005	100%
GAAGAAGAAGA	0.0005	100%
TTTTTTTTAAA	0.0005	100%
TTTAAAAAAAA	0.0005	97.4%
TCTTCTTCTTC	0.0005	100%
TTATTATTATT	0.0005	100%
TTCTTTTTTTT	0.0005	100%
AATAATAATAA	0.0005	100%
AAAAAAAAGAA	0.0005	100%
AGAAGAAGAAG	0.0004	100%
TTTTTGTTTTT	0.0004	100%
AAAAAAAAACA	0.0004	100%
AAAAACAAAAA	0.0004	100%
TGTTTTTTTTT	0.0004	100%
CTTCTTCTTCT	0.0004	100%
GGTGGTGGTGG	0.0004	100%
TCATCATCATC	0.0004	100%
TTTTGTTTTTT	0.0004	100%
CCACCACCACC	0.0004	100%
GTTGTTGTTGT	0.0004	100%
ATTATTATTAT	0.0004	100%
GATGATGATGA	0.0004	100%
AAAAAACAAAA	0.0004	100%
ATAATAATAAT	0.0004	100%
TGGTGGTGGTG	0.0003	100%
CACCACCACCA	0.0003	100%
ACAACAACAAC	0.0003	100%
TATTATTATTA	0.0003	100%
TAATAATAATA	0.0003	100%
TTTTTTAAAAA	0.0003	97.4%
TTTTTAAAAAA	0.0003	94.9%
TTTTTTGTTTT	0.0003	100%
GAGGAAGAGGA	0.0003	100%

**CDS - CDS**	TGCTGCTGCTG	0.0106	100%
	CAGCAGCAGCA	0.0101	100%
	GCTGCTGCTGC	0.0082	100%
	CTGCTGCTGCT	0.0079	100%
	GCAGCAGCAGC	0.0078	100%
	AGCAGCAGCAG	0.0075	100%
	TCCTCCTCCTC	0.0051	100%
	GAGGAGGAGGA	0.0050	100%
	CCTCCTCCTCC	0.0036	100%
	GGAGGAGGAGG	0.0035	100%
	CTCCTCCTCCT	0.0032	100%
	AGGAGGAGGAG	0.0032	100%
	GGCGGCGGCGG	0.0019	94.9%
	CCGCCGCCGCC	0.0017	92.3%
	GCGGCGGCGGC	0.0017	97.4%
	GCCGCCGCCGC	0.0016	92.3%
	CGGCGGCGGCG	0.0011	94.9%
	CGCCGCCGCCG	0.0010	92.3%
	AAGAAGAAGAA	0.0009	100%
	GAAGAAGAAGA	0.0009	100%
	GAGGAAGAGGA	0.0008	100%
	TCCTCTTCCTC	0.0008	100%
	TCTTCTTCTTC	0.0008	100%
	TTCTTCTTCTT	0.0008	100%
	GGTGGTGGTGG	0.0007	100%
	CCACCACCACC	0.0007	100%
	TCCTCCTCTTC	0.0007	100%
	TCTTCCTCCTC	0.0007	100%
	GAAGAGGAGGA	0.0007	100%
	AGAAGAAGAAG	0.0007	100%
	GAGGAGGAAGA	0.0007	100%
	TGGTGGTGGTG	0.0007	100%
	CACCACCACCA	0.0007	100%
	TCATCATCATC	0.0007	100%
	CTTCTTCTTCT	0.0006	100%
	GATGATGATGA	0.0006	100%
	TGTTGCTGCTG	0.0006	100%
	CAGCAGCAACA	0.0006	100%
	GCTGCTGCTGG	0.0006	100%
	CCAGCAGCAGC	0.0005	97.4%
	ACAGCAGCAGC	0.0005	100%
	GCTGCTGCTGT	0.0005	100%
	TGTTGTTGTTG	0.0005	100%
	TGCTGCTGTTG	0.0005	100%
	CAACAGCAGCA	0.0005	100%
	CAACAACAACA	0.0005	100%
	ACCACCACCAC	0.0005	100%
	CATCATCATCA	0.0005	100%
	GTGGTGGTGGT	0.0005	100%
	TTGCTGCTGCT	0.0005	100%
	AGCAGCAGCAA	0.0004	100%
	TGATGATGATG	0.0004	100%
	TGCTGTTGCTG	0.0004	100%
	AGGAAGAGGAG	0.0004	97.4%
	CTCCTCTTCCT	0.0004	97.4%
	TCTTCCTCTTC	0.0004	100%
	GAAGAGGAAGA	0.0004	100%
	CAGCAACAGCA	0.0004	100%
	CTTCCTCCTCC	0.0004	100%
	CTGCTGCTGTT	0.0004	100%
	AACAGCAGCAG	0.0004	100%

	AGAGGAGGAGG	0.0003	100%
	GGAGGAGGAAG	0.0003	100%
	GTTGCTGCTGC	0.0003	100%
	CCTCCTCCTCT	0.0003	100%
	TTCCTCCTCCT	0.0003	100%
	GCAGCAGCAAC	0.0003	100%
	TCTTCTTCCTC	0.0003	100%
	GAGGAAGAAGA	0.0003	100%
	AGGAGGAGGAA	0.0003	100%
	TTGTTGTTGTT	0.0003	100%
	AGCTGCTGCTG	0.0003	100%
	ATCATCATCAT	0.0003	100%
	CTCTTCCTCCT	0.0003	94.9%
	CCTCTTCCTCC	0.0003	94.9%
	CAGCAGCAGCT	0.0003	100%
	CCTCCTCTTCC	0.0003	94.9%
	GGAGGAAGAGG	0.0003	97.4%
	AACAACAACAA	0.0003	100%
	GGAAGAGGAGG	0.0003	97.4%
	AGGAGGAAGAG	0.0003	94.9%
	CTCCTCCTCTT	0.0003	97.4%
	AAGAGGAGGAG	0.0003	97.4%
	GTTGTTGTTGT	0.0003	100%
	TCCTCTTCTTC	0.0003	100%
	ATGATGATGAT	0.0003	100%
	CTCCTCCTCCA	0.0003	97.4%
	GAAGAAGAGGA	0.0003	100%
	TGGAGGAGGAG	0.0003	97.4%
	ACAACAACAAC	0.0003	100%
	GGCTGCTGCTG	0.0002	97.4%
	CTGTTGCTGCT	0.0002	100%
	CAGCAGCAGCC	0.0002	97.4%
	GCTGCTGCAGC	0.0002	100%
	GCTGCAGCAGC	0.0002	100%
	GCAGCAGCAGG	0.0002	92.3%
	AGCAGCAACAG	0.0002	100%
	GCAGCTGCTGC	0.0002	100%
	AAGAAAAAGAA	0.0002	100%
**Introns -Introns**	AAAAAAAAAAA	0.0990	100%
	TTTTTTTTTTT	0.0987	100%
	TGTGTGTGTGT	0.0480	97.4%
	ACACACACACA	0.0478	97.4%
	GTGTGTGTGTG	0.0455	97.4%
	CACACACACAC	0.0453	97.4%
	ATATATATATA	0.0363	100%
	TATATATATAT	0.0363	100%
	AGAGAGAGAGA	0.0167	97.4%
	TCTCTCTCTCT	0.0165	100%
	GAGAGAGAGAG	0.0157	97.4%
	CTCTCTCTCTC	0.0155	97.4%
	GGGGGGGGGGG	0.0034	94.9%
	CCCCCCCCCCC	0.0034	94.9%
	TAAAAAAAAAA	0.0026	100%
	TTTTTTTTTTA	0.0025	100%
	AATAATAATAA	0.0021	100%
	CTTTTTTTTTT	0.0021	100%
	AAAAAAAAAAG	0.0021	100%
	TTATTATTATT	0.0021	100%
	ATAATAATAAT	0.0018	100%
	TTTTTTTTTAA	0.0018	100%
	TTAAAAAAAAA	0.0018	100%

ATTATTATTAT	0.0018	100%
AAAAAAAAAAT	0.0017	100%
ATTTTTTTTTT	0.0017	100%
TAATAATAATA	0.0017	100%
TATTATTATTA	0.0016	100%
TTTATTTATTT	0.0016	100%
AAATAAATAAA	0.0016	100%
CAAAAAAAAAA	0.0014	100%
TTTTTTTTTTG	0.0014	100%
AAAAAAAAAAC	0.0012	100%
GTTTTTTTTTT	0.0012	100%
TTTTTTTTTTC	0.0011	100%
GAAAAAAAAAA	0.0011	100%
TCTTTTTTTTT	0.0010	100%
AAAAAAAAAGA	0.0010	100%
ATTTATTTATT	0.0009	100%
AATAAATAAAT	0.0009	100%
TTTTTTTTAAA	0.0008	100%
TTTAAAAAAAA	0.0008	100%
TATTTATTTAT	0.0007	100%
ATAAATAAATA	0.0007	100%
TTATTTATTTA	0.0007	100%
TAAATAAATAA	0.0007	100%
TTCTTTTTTTT	0.0007	100%
TGTTTTTTTTT	0.0007	100%
AAAAAAAAGAA	0.0007	100%
AAAAAAAAACA	0.0007	100%
TTTTTTTTTCT	0.0006	100%
AGAAAAAAAAA	0.0006	100%
TTTCTTTTTTT	0.0006	100%
AAAAAAAGAAA	0.0006	100%
AATTTTTTTTT	0.0006	100%
AAAAAAAAATT	0.0006	100%
TTTTCTTTTTT	0.0006	100%
AAAAAAGAAAA	0.0006	100%
TTTTTCTTTTT	0.0005	100%
AAAAAGAAAAA	0.0005	100%
ATTAAAAAAAA	0.0005	100%
TTTTTTTTAAT	0.0005	100%
AAAAATAAAAA	0.0005	100%
TTTTTATTTTT	0.0005	100%
TTTTTTTTCTT	0.0005	100%
AAAAAAAAATA	0.0005	100%
AAGAAAAAAAA	0.0005	100%
TATTTTTTTTT	0.0005	100%
TTTTTTTCTTT	0.0005	100%
AAAAAATAAAA	0.0005	100%
AAAGAAAAAAA	0.0005	100%
TTTTATTTTTT	0.0005	100%
TTTTTTCTTTT	0.0004	100%
TTTCTTTCTTT	0.0004	100%
AAAAGAAAAAA	0.0004	100%
AAAGAAAGAAA	0.0004	100%
TTTTTTAAAAA	0.0004	100%
TTTTTAAAAAA	0.0004	100%
AAAAAAATAAA	0.0004	100%
TTTATTTTTTT	0.0004	100%
TTTGTTTGTTT	0.0004	100%
AAACAAACAAA	0.0004	100%
TTTTTGTTTTT	0.0004	100%
ATAAAAAAAAA	0.0004	100%
TTTTTTTTTGT	0.0004	100%

	TTTTTTTTTAT	0.0004	100%
	ACAAAAAAAAA	0.0004	100%
	AAAAACAAAAA	0.0004	100%
	TTGTTTTTTTT	0.0003	100%
	AAAAAAAACAA	0.0003	100%
	TTTTTTATTTT	0.0003	100%
	AAAATAAAAAA	0.0003	100%
	TTTTGTTTTTT	0.0003	100%
	TTGTTGTTGTT	0.0003	100%
	AAAAAACAAAA	0.0003	100%
	TTTTTTTAAAT	0.0003	100%
	AATAAAAAAAA	0.0003	100%
	ATTTAAAAAAA	0.0003	100%
	AACAACAACAA	0.0003	100%
	TTTTTTTTATT	0.0003	100%
**Intergenic – Intergenic**	TTTTTTTTTTT	0.0837	100%
	AAAAAAAAAAA	0.0835	100%
	TGTGTGTGTGT	0.0452	97.4%
	ACACACACACA	0.0440	97.4%
	GTGTGTGTGTG	0.0428	97.4%
	TATATATATAT	0.0422	100%
	ATATATATATA	0.0422	100%
	CACACACACAC	0.0417	97.4%
	AGAGAGAGAGA	0.0194	100%
	TCTCTCTCTCT	0.0192	100%
	GAGAGAGAGAG	0.0182	100%
	CTCTCTCTCTC	0.0181	100%
	CCCCCCCCCCC	0.0032	89.7%
	GGGGGGGGGGG	0.0030	92.3%
	TTATTATTATT	0.0029	100%
	AATAATAATAA	0.0028	100%
	ATAATAATAAT	0.0025	100%
	ATTATTATTAT	0.0025	100%
	TATTATTATTA	0.0023	100%
	TAATAATAATA	0.0023	100%
	TTTTTTTTTTA	0.0019	100%
	TAAAAAAAAAA	0.0019	100%
	TTTATTTATTT	0.0018	100%
	AAATAAATAAA	0.0018	100%
	CTTTTTTTTTT	0.0017	100%
	AAAAAAAAAAG	0.0017	100%
	TTAAAAAAAAA	0.0014	100%
	AAAAAAAAAAT	0.0014	100%
	TTTTTTTTTAA	0.0014	100%
	ATTTTTTTTTT	0.0013	100%
	CAAAAAAAAAA	0.0012	100%
	TTTTTTTTTTG	0.0012	100%
	ATTTATTTATT	0.0011	100%
	AATAAATAAAT	0.0010	100%
	GAAAAAAAAAA	0.0010	100%
	TTTTTTTTTTC	0.0010	100%
	AAAAAAAAAAC	0.0010	100%
	GTTTTTTTTTT	0.0010	100%
	TCTTTTTTTTT	0.0009	100%
	AAAAAAAAAGA	0.0009	100%
	TATTTATTTAT	0.0009	100%
	ATAAATAAATA	0.0009	100%
	TTATTTATTTA	0.0008	100%
	TAAATAAATAA	0.0008	100%
	AAAGAAAGAAA	0.0007	100%
	TTTCTTTCTTT	0.0007	100%

	AAAAAAAAGAA	0.0007	100%
	TTCTTTTTTTT	0.0006	100%
	TTTCTTTTTTT	0.0006	100%
	AAAAAAAGAAA	0.0006	100%
	TTTAAAAAAAA	0.0006	100%
	TTTTTTTTAAA	0.0006	100%
	AGAAAAAAAAA	0.0006	100%
	TTTTTTTTTCT	0.0006	100%
	AAAAAAAAACA	0.0006	100%
	TGTTTTTTTTT	0.0006	100%
	TTTTCTTTTTT	0.0006	100%
	AAAAAAGAAAA	0.0006	100%
	TTTTTCTTTTT	0.0005	100%
	AAAAAGAAAAA	0.0005	100%
	TTTTTATTTTT	0.0005	100%
	AATTTTTTTTT	0.0005	100%
	AAAAATAAAAA	0.0005	100%
	AAAAAAAAATT	0.0005	100%
	AAGAAAAAAAA	0.0005	100%
	AAAGAAAAAAA	0.0005	100%
	AAAAAATAAAA	0.0005	100%
	TTTTATTTTTT	0.0005	100%
	TTTTTTTCTTT	0.0005	100%
	TTTTTTTTCTT	0.0004	100%
	TTTTTTTTAAT	0.0004	100%
	TTTTTTCTTTT	0.0004	100%
	ATTAAAAAAAA	0.0004	100%
	AAACAAACAAA	0.0004	100%
	AAAAGAAAAAA	0.0004	100%
	TTTGTTTGTTT	0.0004	100%
	AGAAAGAAAGA	0.0004	89.7%
	TCTTTCTTTCT	0.0004	87.2%
	AAAAAAAAATA	0.0004	100%
	GAAAGAAAGAA	0.0004	89.7%
	TTCTTTCTTTC	0.0004	87.2%
	TATTTTTTTTT	0.0004	100%
	TTTATTTTTTT	0.0004	100%
	AAGAAAGAAAG	0.0004	87.2%
	AAAAAAATAAA	0.0004	100%
	CTTTCTTTCTT	0.0004	87.2%
	TTTTTTAAAAA	0.0004	97.4%
	TTTTTAAAAAA	0.0004	97.4%
	AACAACAACAA	0.0004	100%
	TTGTTGTTGTT	0.0004	97.4%
	TTTTTTATTTT	0.0003	100%
	AAAAACAAAAA	0.0003	100%
	TTATTTATTTT	0.0003	100%
	AAAATAAAAAA	0.0003	100%
	TTTTTGTTTTT	0.0003	100%
	AAAATAAATAA	0.0003	100%
	ACAAAAAAAAA	0.0003	100%
	TTTTTTTTTGT	0.0003	100%
	ATAAAAAAAAA	0.0003	100%
	TTTTTTTTTAT	0.0003	100%
**Introns - Intergenic**	AAAAAAAAAAA	0.0884	
	TTTTTTTTTTT	0.0883	
	TGTGTGTGTGT	0.0456	
	ACACACACACA	0.0448	
	GTGTGTGTGTG	0.0432	
	CACACACACAC	0.0425	
	ATATATATATA	0.0395	

TATATATATAT	0.0395
AGAGAGAGAGA	0.0181
TCTCTCTCTCT	0.0178
GAGAGAGAGAG	0.0170
CTCTCTCTCTC	0.0168
CCCCCCCCCCC	0.0042
GGGGGGGGGGG	0.0041
AATAATAATAA	0.0025
TTATTATTATT	0.0025
ATAATAATAAT	0.0022
TAAAAAAAAAA	0.0022
TTTTTTTTTTA	0.0022
ATTATTATTAT	0.0022
TAATAATAATA	0.0020
TATTATTATTA	0.0020
CTTTTTTTTTT	0.0019
AAAAAAAAAAG	0.0018
TTTATTTATTT	0.0017
AAATAAATAAA	0.0016
TTAAAAAAAAA	0.0016
TTTTTTTTTAA	0.0016
AAAAAAAAAAT	0.0016
ATTTTTTTTTT	0.0015
CAAAAAAAAAA	0.0013
TTTTTTTTTTG	0.0013
AAAAAAAAAAC	0.0011
TTTTTTTTTTC	0.0011
GAAAAAAAAAA	0.0011
GTTTTTTTTTT	0.0011
TCTTTTTTTTT	0.0010
AAAAAAAAAGA	0.0010
ATTTATTTATT	0.0009
AATAAATAAAT	0.0009
TATTTATTTAT	0.0008
ATAAATAAATA	0.0008
TTATTTATTTA	0.0008
TAAATAAATAA	0.0008
TTTAAAAAAAA	0.0007
TTTTTTTTAAA	0.0007
TTCTTTTTTTT	0.0007
AAAAAAAAGAA	0.0007
AAAAAAAAACA	0.0006
TGTTTTTTTTT	0.0006
AGAAAAAAAAA	0.0006
TTTTTTTTTCT	0.0006
TTTCTTTTTTT	0.0006
AAAAAAAGAAA	0.0006
AATTTTTTTTT	0.0006
AAAAAAAAATT	0.0006
TTTTCTTTTTT	0.0006
AAAAAAGAAAA	0.0005
TTTCTTTCTTT	0.0005
AAAGAAAGAAA	0.0005
TTTTTCTTTTT	0.0005
AAAAAGAAAAA	0.0005
ATTAAAAAAAA	0.0005
TTTTTTTTAAT	0.0005
TTTTTATTTTT	0.0005
AAAAATAAAAA	0.0005
AAGAAAAAAAA	0.0005
TTTTTTTTCTT	0.0005
AAAAAAAAATA	0.0005

	AAAAAATAAAA	0.0005
	TTTTATTTTTT	0.0005
	AAAGAAAAAAA	0.0004
	TTTTTTTCTTT	0.0004
	TATTTTTTTTT	0.0004
	TTTTTTCTTTT	0.0004
	AAAAGAAAAAA	0.0004
	TTTTTTAAAAA	0.0004
	TTTTTAAAAAA	0.0004
	TTTATTTTTTT	0.0004
	AAAAAAATAAA	0.0004
	AAACAAACAAA	0.0004
	TTTGTTTGTTT	0.0004
	ATAAAAAAAAA	0.0004
	TTTTTTTTTAT	0.0004
	TTTTTTTTTGT	0.0003
	TTTTTGTTTTT	0.0003
	ACAAAAAAAAA	0.0003
	AAAAACAAAAA	0.0003
	TTTTTTATTTT	0.0003
	AAAATAAAAAA	0.0003
	TTGTTGTTGTT	0.0003
	AACAACAACAA	0.0003
	AAAAAAAACAA	0.0003
	TTATTTATTTT	0.0003
	TTGTTTTTTTT	0.0003
	AATAAAAAAAA	0.0003
	AAAATAAATAA	0.0003
	TTTTTTTTATT	0.0003
	TCTTTCTTTCT	0.0003
**Exons - Introns**	TTTTTTTTTTT	0.1002
	AAAAAAAAAAA	0.0999
	ACACACACACA	0.0344
	TGTGTGTGTGT	0.0338
	CACACACACAC	0.0328
	GTGTGTGTGTG	0.0323
	ATATATATATA	0.0223
	TATATATATAT	0.0222
	AGAGAGAGAGA	0.0096
	TCTCTCTCTCT	0.0095
	GAGAGAGAGAG	0.0090
	CTCTCTCTCTC	0.0090
	GGGGGGGGGGG	0.0067
	CCCCCCCCCCC	0.0063
	TAAAAAAAAAA	0.0025
	TTTTTTTTTTA	0.0024
	CTTTTTTTTTT	0.0022
	AAAAAAAAAAG	0.0022
	TTTTTTTTTAA	0.0017
	TTAAAAAAAAA	0.0017
	AAAAAAAAAAT	0.0015
	ATTTTTTTTTT	0.0014
	CAGCAGCAGCA	0.0014
	TGCTGCTGCTG	0.0014
	TTATTATTATT	0.0014
	AATAATAATAA	0.0013
	GAAAAAAAAAA	0.0012
	TTTTTTTTTTC	0.0012
	AGCAGCAGCAG	0.0012
	TTTTTTTTTTG	0.0012
	CTGCTGCTGCT	0.0012

GCAGCAGCAGC	0.0012
CAAAAAAAAAA	0.0012
ATAATAATAAT	0.0011
GCTGCTGCTGC	0.0011
ATTATTATTAT	0.0011
AAAAAAAAAAC	0.0011
GTTTTTTTTTT	0.0011
TAATAATAATA	0.0010
TATTATTATTA	0.0010
TCTTTTTTTTT	0.0010
AAAAAAAAAGA	0.0010
TTTATTTATTT	0.0008
AAATAAATAAA	0.0008
TTTTTCTTTTT	0.0007
TTTTTTTTAAA	0.0007
AGAAAAAAAAA	0.0007
AAAAAGAAAAA	0.0007
TTTAAAAAAAA	0.0007
TTTTTTTTTCT	0.0007
TTTCTTTTTTT	0.0007
TTTTCTTTTTT	0.0007
AAAAAAGAAAA	0.0007
AAAAAAAGAAA	0.0007
TTCTTTTTTTT	0.0007
AAAAAAAAGAA	0.0007
AAAAAAAAACA	0.0006
TGTTTTTTTTT	0.0006
AAGAAAAAAAA	0.0006
TTTTTTTTCTT	0.0006
TTTTTTTCTTT	0.0006
AAAGAAAAAAA	0.0006
TTTTTTCTTTT	0.0006
AAAAGAAAAAA	0.0006
TTGTTGTTGTT	0.0006
AACAACAACAA	0.0005
GAGGAGGAGGA	0.0005
TCCTCCTCCTC	0.0005
TTTTTGTTTTT	0.0005
AAAAACAAAAA	0.0005
GGAGGAGGAGG	0.0004
CCTCCTCCTCC	0.0004
TTTTTTAAAAA	0.0004
ATTAAAAAAAA	0.0004
TTTTTTTTAAT	0.0004
TTTTTAAAAAA	0.0004
TTTTTATTTTT	0.0004
AAAAATAAAAA	0.0004
AGGAGGAGGAG	0.0004
TTTTATTTTTT	0.0004
TTTGTTTGTTT	0.0004
CTCCTCCTCCT	0.0004
TTTTGTTTTTT	0.0004
ATTTATTTATT	0.0004
AAAAAATAAAA	0.0004
TGTTGTTGTTG	0.0004
AATAAATAAAT	0.0004
AAACAAACAAA	0.0004
AAAAAACAAAA	0.0004
CAACAACAACA	0.0004
TTTCTTTCTTT	0.0004
AATTTTTTTTT	0.0004
TATTTTTTTTT	0.0004

	AAAGAAAGAAA	0.0004
	AAAAAAAAATT	0.0004
	TTTATTTTTTT	0.0004
	AAAAAAAAATA	0.0004
	TTTTTTGTTTT	0.0004
	AAAAAAATAAA	0.0004
**Exons - CDS**	TGCTGCTGCTG	0.0093
	CAGCAGCAGCA	0.0091
	GCTGCTGCTGC	0.0073
	CTGCTGCTGCT	0.0071
	GCAGCAGCAGC	0.0071
	AGCAGCAGCAG	0.0069
	GAGGAGGAGGA	0.0044
	TCCTCCTCCTC	0.0044
	CCTCCTCCTCC	0.0032
	GGAGGAGGAGG	0.0032
	AGGAGGAGGAG	0.0030
	CTCCTCCTCCT	0.0029
	GGCGGCGGCGG	0.0019
	GCGGCGGCGGC	0.0018
	CCGCCGCCGCC	0.0018
	GCCGCCGCCGC	0.0017
	AAAAAAAAAAA	0.0014
	TTTTTTTTTTT	0.0014
	CGGCGGCGGCG	0.0012
	GGGGGGGGGGG	0.0011
	CGCCGCCGCCG	0.0011
	ACACACACACA	0.0010
	CACACACACAC	0.0010
	AAGAAGAAGAA	0.0009
	GTGTGTGTGTG	0.0009
	TGTGTGTGTGT	0.0009
	GAAGAAGAAGA	0.0008
	TTCTTCTTCTT	0.0008
	TCTTCTTCTTC	0.0008
	AGAAGAAGAAG	0.0007
	TGTTGTTGTTG	0.0007
	GGTGGTGGTGG	0.0007
	CCACCACCACC	0.0007
	TCATCATCATC	0.0006
	GAGGAAGAGGA	0.0006
	CCCCCCCCCCC	0.0006
	CTTCTTCTTCT	0.0006
	TCCTCTTCCTC	0.0006
	CAACAACAACA	0.0006
	TGGTGGTGGTG	0.0006
	GATGATGATGA	0.0006
	CACCACCACCA	0.0006
	TTGTTGTTGTT	0.0006
	TCTCTCTCTCT	0.0006
	TCCTCCTCTTC	0.0005
	TCTTCCTCCTC	0.0005
	GAAGAGGAGGA	0.0005
	TGTTGCTGCTG	0.0005
	GAGGAGGAAGA	0.0005
	AACAACAACAA	0.0005
	AGAGAGAGAGA	0.0005
	CAGCAGCAACA	0.0005
	CTCTCTCTCTC	0.0005
	CATCATCATCA	0.0005
	ACAGCAGCAGC	0.0005

GCTGCTGCTGT	0.0005
GCTGCTGCTGG	0.0004
GAGAGAGAGAG	0.0004
TGATGATGATG	0.0004
TGCTGCTGTTG	0.0004
CCAGCAGCAGC	0.0004
CAACAGCAGCA	0.0004
GTGGTGGTGGT	0.0004
GTTGTTGTTGT	0.0004
ACCACCACCAC	0.0004
TTGCTGCTGCT	0.0004
AGCAGCAGCAA	0.0004
ACAACAACAAC	0.0004
TTTTTCTTTTT	0.0004
ATCATCATCAT	0.0004
AAAAAGAAAAA	0.0004
TGCTGTTGCTG	0.0004
CAGCAACAGCA	0.0003
AGGAAGAGGAG	0.0003
ATGATGATGAT	0.0003
CTCCTCTTCCT	0.0003
AACAGCAGCAG	0.0003
CTGCTGCTGTT	0.0003
CTTCCTCCTCC	0.0003
GTTGCTGCTGC	0.0003
TCTTCCTCTTC	0.0003
GCAGCAGCAAC	0.0003
GAAGAGGAAGA	0.0003
AGAGGAGGAGG	0.0003
GGAGGAGGAAG	0.0003
CCTCCTCCTCT	0.0003
TTCCTCCTCCT	0.0003
AGGAGGAGGAA	0.0003
AGCTGCTGCTG	0.0003
AAGAAAAAGAA	0.0003
TTCTTTTTCTT	0.0003
CAGCAGCAGCT	0.0003
CTCTTCCTCCT	0.0003
CCTCTTCCTCC	0.0003
GAGGAAGAAGA	0.0003
TCTTCTTCCTC	0.0003
GGAGGAAGAGG	0.0003
GGAAGAGGAGG	0.0003
CCTCCTCTTCC	0.0003

Significance of Correlation Values

To confirm that observed (high) correlation values (> 0.4) are not a product of random chance, we used Markov models of different orders to generate randomly shuffled sequences with comparable sequence properties (e.g. G/C content) and compared the correlation results with the correlations found on real sequences.

The results for correlations of completely random sequences (Markov order zero) are shown in Fig. SX1 and SX2. The mean values and standard deviations (μ=-0.199, σ=0.001 for k=10; μ=0.00, σ=0.01 for k=7) clearly show that correlations values above 0.4 are extremely improbable for random sequences.

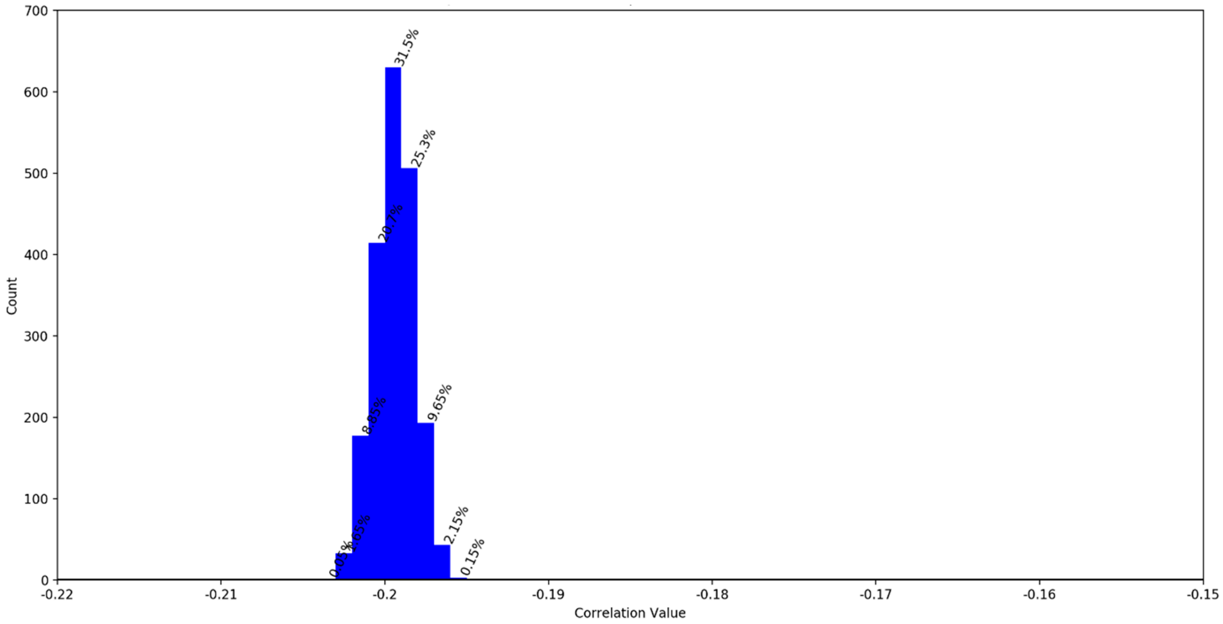

Figure S2. Markov Simulation (order: zero, k=10). Correlation results for 2000 Markov simulations of order zero, sequence length was 1Mbp for each independent simulation.

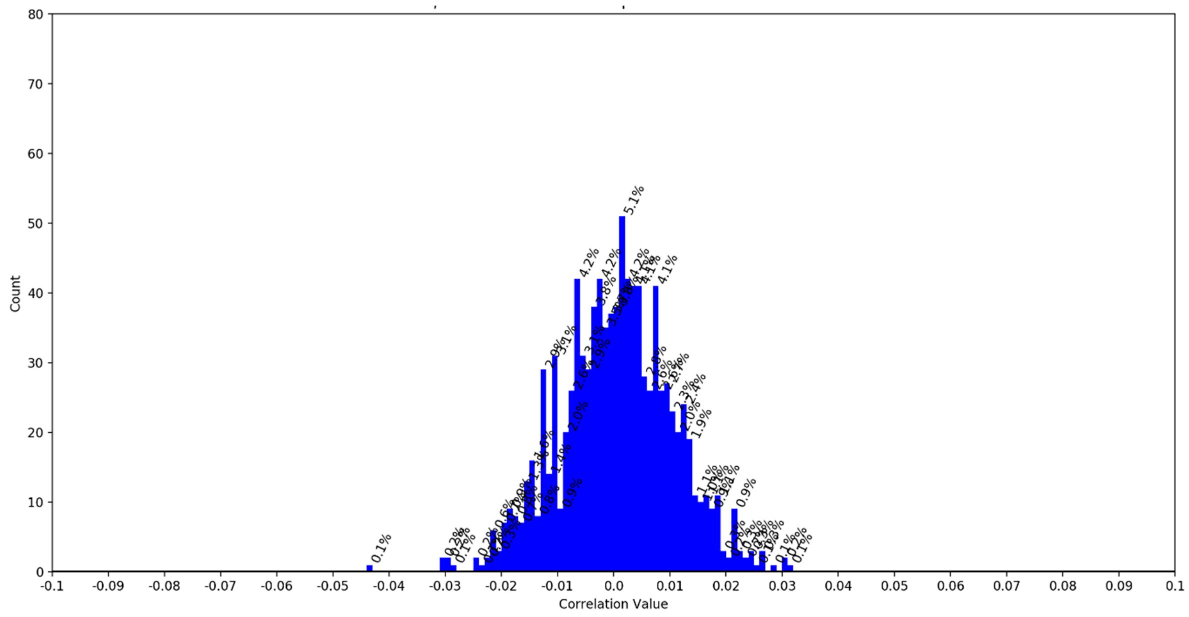

Figure S3. Markov Simulation (order: zero, k=7). Correlation results for 1000 Markov simulations of order zero, sequence length was 1Mbp for each independent simulation.

Simulations for higher Markov orders are shown in Fig. SX3, SX4. To create these results, we correlated *k*-mer spectra of random sequences (different Markov orders, based on the analyzed *Animalia* genomes) with the spectra of the *Animalia* genomes analyzed in this article (see table 1). According to [C2], Markov models of low orders (<3) are able to reproduce significant properties of DNA word spectra. The results again illustrate that correlation values higher than 0.4 are extremely improbable to be the product of randomness.

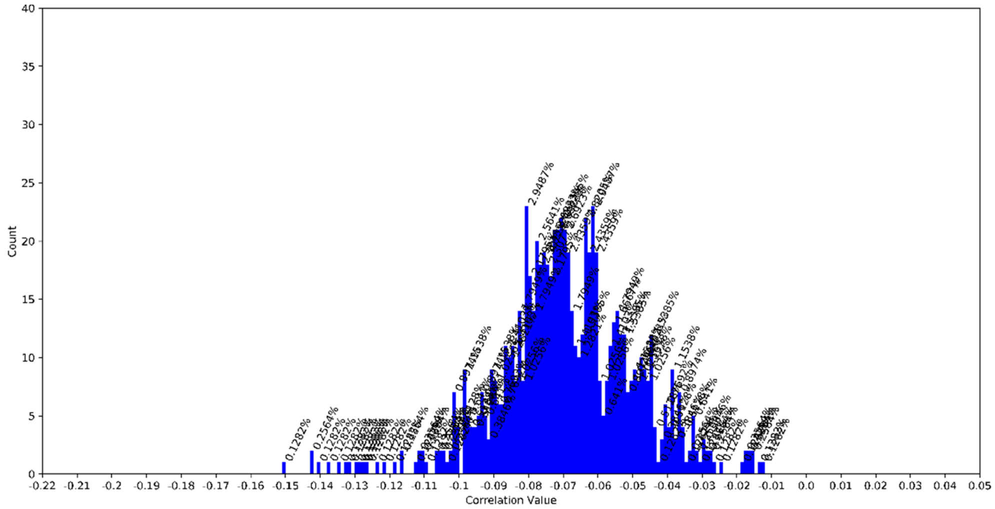

Figure S4. Markov Simulation (order: 1, k=7). Correlation results for 780 Markov simulations of order zero, sequence length was 1Mbp for each independent simulation.

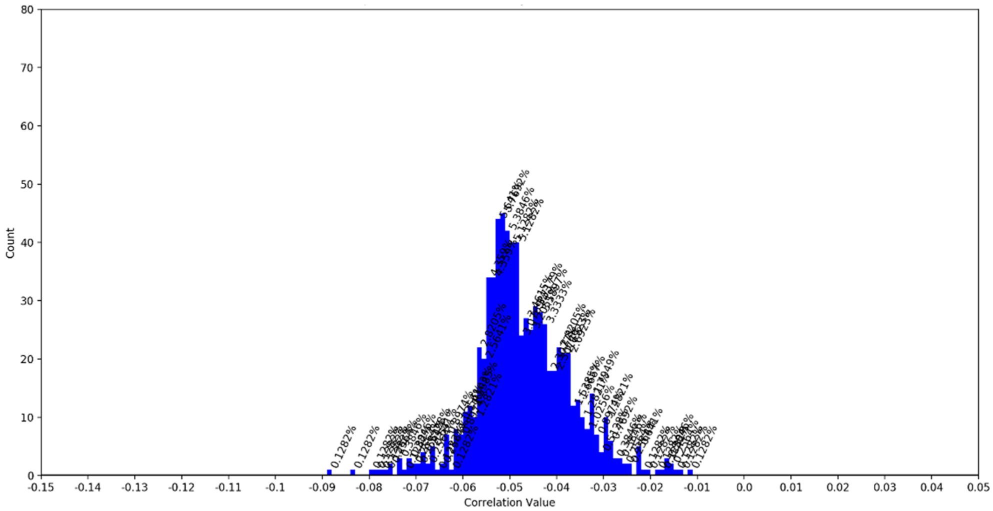

Figure S5. Markov Simulation (order: 2, k=7). Correlation results for 780 Markov simulations of order zero, sequence length was 1Mbp for each independent simulation.
